# Supplementary material for: Design, Synthesis and Various Bioactivity of Acylhydrazone-Containing Matrine Analogues
Source: Molecules. 2023 May 18;28(10):4163. doi: 10.3390/molecules28104163 (PMC10221637; doi:10.3390/molecules28104163)
Supplement: Supplementary file 1 [file molecules-28-04163-s001.zip › molecules-2381587-supplementary.pdf]

Design, Synthesis and Various Bioactivity of Acylhydrazone-Containing Matrine Analogues

Wanjun Ni, Hongjian Song, Lizhong Wang, Yuxiu Liu * and Qingmin Wang *

State Key Laboratory of Elemento-Organic Chemistry, Research Institute of Elemento-Organic Chemistry, College of Chemistry, Frontiers Science Center for New Organic Matter, Nankai University, Tianjin 300071, China; 2120140672@mail.nankai.edu.cn (W.N.); songhongjian@nankai.edu.cn (H.S.); wlzhong@nankai.edu.cn (L.W.)

* Correspondence: liuyuxiu@nankai.edu.cn (Y.L.); wangqm@nankai.edu.cn (Q.W.); Tel.: +86-(0)22-23503792 (Y.L.); +86-(0)22-23503952 (Q.W.)

supporting information

1. Data of intermediate **A**–**D** and target compounds **1**–**45**………………………….. 2
2. NMR spectra of intermediates **A**–**D** and target compounds **1**–**45**……………..…22
3. Bioassay methods for anti-TMV, insecticidal and fungicidal activities………… 47
4. Insecticidal activity (Table S1)…………………………………………………..…50
5. Fungicidal activity (Table S2)……………………………………………………52
6. References……………………………………………………………………..55

1. Data of intermediate A–E and target compounds 1–45

Methyl (41S,7aS,13aR,13bR)-10-oxododecahydro-1H,5H,8H-dipyrido[2,1-f:3',2',1'-ij][1,6]naphthyridine-11-carboxylate (Methyl matrine-14-carboxylate, **A**)[S1]

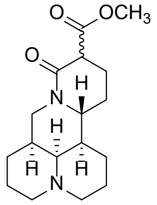

Yellow oil. ^{1}H NMR (400 MHz, $CDCl_3$) δ 4.49 – 4.32 (m, 1H), 3.91 – 3.83 (m, 1H), 3.76 (s, 1.5H), 3.74 (s, 1.5H), 3.48 (s, 0.5H), 3.28 (dd, J = 9.6, 2.8 Hz, 0.5H), 3.10 (t, J = 12.8 Hz, 1H), 2.86 – 2.70 (m, 2H), 2.22-2.11 (m, 1H), 2.11 – 1.76 (m, 6H), 1.73 – 1.35 (m, 10H).

(41S,7aS,13aR,13bR)-10-Oxododecahydro-1H,5H,8H-dipyrido[2,1-f:3',2',1'-ij][1,6] naphthyridine-11-carbohydrazide (Matrine-14-carbohydrazide, **B**)

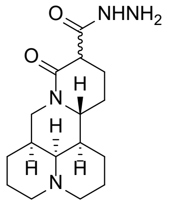

Yellow oil. ^{1}H NMR (400 MHz, $CDCl_3$) δ 4.39 – 4.01 (m, 1H), 3.92 – 3.79 (m, 1H), 3.59 (brs, 2H, NH_2), 3.28 (s, 0.5H), 3.16 – 3.02 (m, 2H), 2.81 (dd, J = 11.2, 10.8 Hz, 2H), 2.42 – 2.37 (m, 0.5H), 2.11 – 1.86 (m, 6H), 1.73 – 1.35 (m, 10H). ^{13}C NMR (100 MHz, DMSO-d_6) δ 169.5, 169.4, 165.9, 165.4, 63.1, 62.8, 56.6, 56.5, 52.7, 52.5, 47.5, 46.8, 42.8, 41.9, 41.6, 40.9, 35.3, 34.9, 27.4, 27.4, 25.9, 25.8, 25.2, 25.1, 22.5, 21.9, 20.7, 20.6, 20.2, 14.0, 13.6. HRMS (ESI) calcd for $C_{16}H_{27}N_4O_2$ $[M+H]^+$ 307.2129, found 307.2133.

4-((1R,3aS,3a1S,10aR)-Decahydro-1H,4H-pyrido[3,2,1-ij][1,6]naphthyridin-1-yl)butanoic acid (Matric acid, **C**)[S2]

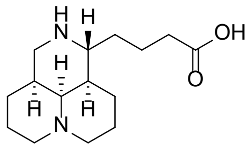

The mixture of matrine (2.48 g, 0.01 mol) and 10% NaOH solution (40 mL, 0.1 mol) was refluxed and the process was monitored by TLC. After the reaction was complete, the mixture was acidified with 20% sulfuric acid to pH = 7-8, and then condensed in reduced pressure. The residue was resolved in methanol and filtered when still hot. The filtrate was condensed to a quarter amount of the original amount, and then acetone was added and stirred vigorously. The resulted white solid **C** was collected by filtration. Mp 198 – 199 °C. ^{1}H NMR (400 MHz, D_2O) δ 3.23– 3.17 (m, 1H), 3.09 (t, J = 12.8 Hz, 1H), 2.93 – 2.80 (m, 1H), 2.71 (t, J = 12.0 Hz, 2H), 2.40 (s,

1H), 2.13 – 1.30 (m, 18H).

Benzyl 4-((1R,3aS,3a1S,10aR)-2-benzyldecahydro-1H,4H-pyrido[3,2,1-ij][1,6]naphthyridin-1-yl)butanoate (Benzyl *N*-benzyl-matrine-1-butanoate, **D**)[S2]

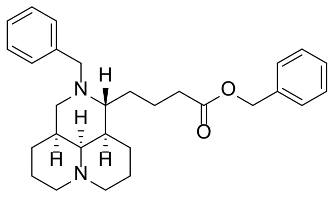

The solution of matric acid (**C**) (2.66 g, 0.01 mol) and K_2CO_3 in DMF (20 mL) was heated to 75 ºC, and then benzyl chloride (5 mL) was added. After the reaction was complete indicated by TLC, the mixture was poured into water (100 mL) and extracted with petroleum ether. The organic layer was washed with brine, dried over Na_2SO_4, filtered, and concentrated to give D as a white solid. Mp 73.5 – 74.0 ºC. ^{1}H NMR (400 MHz, $CDCl_3$) δ 7.42 – 7.20 (m, 10H), 5.08 (s, 2H, PhC**H**$_2$O), 4.07 (d, J = 12.0 Hz, 1H, PhC**H**$_2$N), 3.08 (d, J = 12.0 Hz, 1H, PhC**H**$_2$N), 2.86 – 2.75 (m, 3H), 2.60 (t, J = 12.0 Hz, 1H), 2.35 – 2.31 (m, 3H), 2.03 (s, 1H), 1.93 – 1.51 (m, 11H), 1.50 – 1.25 (m, 5H).

(41S,7aS,13aR,13bR)-N'-((E)-2,4-Dimethoxybenzylidene)-10-oxododecahydro-1H,5H,8H-dipyrido[2,1-f:3',2',1'-ij][1,6]naphthyridine-11-carbohydrazide (2,4-dimethoxybenzaldehyde matrine-14-carbohydrazone, **1**)

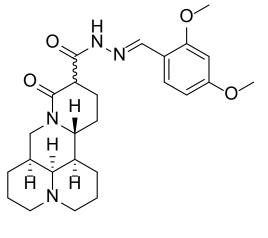

Light yellow solid 0.43 g, yield 60%. Mp 166−167 ºC. ^{1}H NMR (400 MHz, DMSO-*d*$_6$) *δ* 11.36 –11.16 (4s, NH), 8.45 – 8.19 (4s, CH=N), 7.79 – 7.55 (m, 1H), 6.66 – 6.53 (m, 2H), 4.16 (s, 1H), 3.85 – 3.81 (m, 7H), 3.09 – 2.75 (m, 4H), 2.33 – 1.29 (m, 17H). HRMS（ESI）calcd for $C_{25}H_{35}N_4O_4$ $[M+H]^+$ 455.2653, found 455.2660.

(41S,7aS,13aR,13bR)-N'-((E)-2-Methoxybenzylidene)-10-oxododecahydro-1H,5H,8H-dipyrido[2,1-f:3',2',1'-ij][1,6]naphthyridine-11-carbohydrazide (2-methoxybenzaldehyde matrine-14-carbohydrazone, **2**)

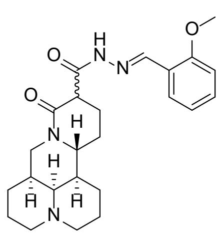

Yellow solid 0.40 g, yield 59%. Mp 170−171 ºC. ^{1}H NMR (400 MHz, DMSO-*d*$_6$) *δ* 11.47 – 11.27 (4s, 1H, NH), 8.55 – 8.28 (4s, 1H,

CH=N), 7.80 – 7.85 (m, 1H), 7.42 – 7.38 (m, 1H), 7.10 – 6.93 (m, 2H), 4.15 – 4.11 (m, 1H), 3.85 – 3.83 (m, 4H), 3.25 – 2.71 (m, 4H), 2.18 – 1.86 (m, 7H), 1.64 – 1.22 (m, 10H). HRMS（ESI）calcd for $C_{24}H_{33}N_4O_3$ $[M+H]^+$ 425.2547, found 425.2551.

(41S,7aS,13aR,13bR)-N'-((E)-4-methoxybenzylidene)-10-oxododecahydro-1H,5H,8H-dipyrido[2,1-f:3',2',1'-ij][1,6]naphthyridine-11-carbohydrazide

(4-Methoxybenzaldehyde matrine-14-carbohydrazone, **3**)

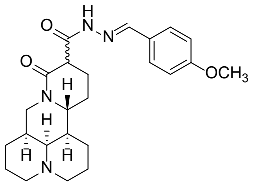

Light yellow solid 0.45 g, yield 66%. Mp 181−182 ºC. ^{1}H NMR (400 MHz, DMSO-d_6) δ 11.35 – 11.20 (4s, 1H, NH), 8.16 – 7.89 (4s, 1H, CH=N), 7.66 – 7.60 (m, 2H), 7.02 – 6.96 (m, 2H), 4.25 – 4.12 (m, 1H), 3.83 – 3.80 (m, 4H), 3.35 – 2.97 (m, 2H), 2.85 – 2.73 (m, 2H), 2.18 – 1.81 (m, 7H), 1.61 – 1.37 (m, 10H). HRMS（ESI）calcd for $C_{24}H_{33}N_4O_3$ $[M+H]^+$ 425.2547, found 425.2554.

(41S,7aS,13aR,13bR)-N'-((E)-4-Methylbenzylidene)-10-oxododecahydro-1H,5H,8H-dipyrido[2,1-f:3',2',1'-ij][1,6]naphthyridine-11-carbohydrazide

(4-methylbenzaldehyde matrine-14-carbohydrazone, **4**)

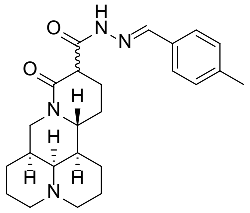

Light yellow solid 0.42 g, yield 70%. Mp 187−188 ºC. ^{1}H NMR (400 MHz, DMSO-d_6) δ 11.46 –11.29 (4s, 1H, NH), 8.19 – 7.83 (m, 1H, CH=N), 7.60 – 7.53 (m, 2H), 7.30 – 7.21 (m, 2H), 4.20 – 4.13 (m, 1H), 3.84 – 3.78 (s, 1H), 3.25 – 2.93 (m, 2H), 2.79 – 2.73 (m, 2H), 2.36 – 2.18 (m, 3H), 2.18 – 1.22 (m, 17H). HRMS（ESI）calcd for $C_{24}H_{33}N_4O_2$ $[M+H]^+$ 409.2598, found 409.2605.

(41S,7aS,13aR,13bR)-N'-((E)-4-(Dimethylamino)benzylidene)-10-oxododecahydro-1H,5H,8H-dipyrido[2,1-f:3',2',1'-ij][1,6]naphthyridine-11-carbohydrazide

(4-dimethylbenzaldehyde matrine-14-carbohydrazone, **5**)

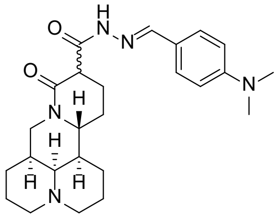

Red solid 0.26 g, yield 75%. Mp 163−164 ºC. ^{1}H NMR (400 MHz, DMSO-d_6) δ 11.26 – 11.04 (4s, 1H, NH), 8.07 – 7.81

(m, 1H, CH=N), 7.50 – 7.45 (m, 2H), 6.74 – 6.68 (m, 2H), 4.18 – 4.11 (m, 1H), 3.88 – 3.73 (s, 1H), 3.29 – 2.97 (m, 1H), 2.96 (s, 6H), 2.83 – 2.75 (m, 2H), 2.18 – 1.86 (m, 7H), 1.57 – 1.16 (m, 10H). HRMS（ESI）calcd for $C_{25}H_{36}N_5O_2$ $[M+H]^+$ 438.2864, found 438.2864.

(41S,7aS,13aR,13bR)-N'-((E)-[1,1'-Biphenyl]-4-ylmethylene)-10-oxododecahydro-1H,5H,8H-dipyrido[2,1-f:3',2',1'-ij][1,6]naphthyridine-11-carbohydrazide (4-phenylbenzaldehyde matrine-14-carbohydrazone, **6**)

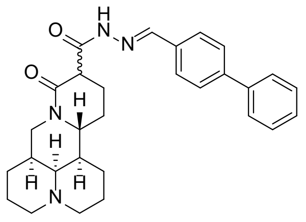

Yellow solid 0.52 g, yield 69%. Mp 190−191 ºC. ^1H NMR (400 MHz, DMSO-d_6) δ 11.52 – 11.37 (4s, 1H, NH), 8.26 – 7.80 (4s, 1H, CH=N), 7.77 – 7.72 (m, 6H), 7.51 –7.39 (m, 3H), 4.23 – 4.13 (m, 1.2H), 3.84 (brs, 1.2H), 3.01 – 2.71 (m, 3.6H), 2.23 – 1.12 (m, 17H). HRMS（ESI）calcd for $C_{29}H_{35}N_4O_2$ $[M+H]^+$ 471.2755, found 471.2759.

(41S,7aS,13aR,13bR)-N'-((E)-2-Hydroxybenzylidene)-10-oxododecahydro-1H,5H,8H-dipyrido[2,1-f:3',2',1'-ij][1,6]naphthyridine-11-carbohydrazide (2-hydroxybenzaldehyde matrine-14-carbohydrazone, **7**)

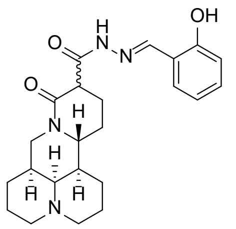

Light yellow solid 0.6 g, yield 65%. Mp 169−170 ºC. ^1H NMR (400 MHz, DMSO-d_6) δ 11.77 (s, 1H, OH), 11.37 − 11.13 (4s, 1H, NH), 8.42 – 8.24 (m, 1H, CH=N), 7.64 – 7.50 (m, 1H), 7.30 – 7.21 (m, 1H), 6.93 – 6.81 (m, 2H), 4.20 – 4.12 (m, 1H), 3.83 – 3.81 (m, 1H), 3.31 – 3.13 (m, 1H) 3.02 – 2.95 (m, 1H), 2.82 – 2.67 (m, 2H), 2.22 – 1.75 (m, 7H), 1.66 – 1.16 (m, 10H). HRMS（ESI）calcd for $C_{23}H_{31}N_4O_3$ $[M+H]^+$ 411.2391, found 411.2395.

(41S,7aS,13aR,13bR)-N'-((E)-3-Hydroxybenzylidene)-10-oxododecahydro-1H,5H,8H-dipyrido[2,1-f:3',2',1'-ij][1,6]naphthyridine-11-carbohydrazide (3-hydroxybenzaldehyde matrine-14-carbohydrazone, **8**)

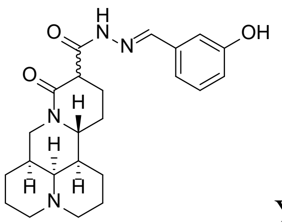

Yellow solid 0.29 g, yield 66%. Mp 167−168 ºC. ^{1}H NMR (400 MHz, DMSO-d_6) δ 11.51 − 11.30 (4 s, 1H, NH), 9.56 (s, 1H, OH), 8.13 – 7.87 (4 s, 1H, CH=N), 7.25 – 6.81 (m, 4H), 4.17 (s, 1H), 3.96 – 3.79 (m, 1H), 3.37 – 2.96 (m, 2H), 2.83 – 2.68 (m, 2H), 2.18 – 1.12 (m, 17H). HRMS（ESI）calcd for $C_{23}H_{31}N_4O_3$ $[M+H]^+$ 411.2391, found 411.2390.

(41S,7aS,13aR,13bR)-N'-((E)-4-Hydroxy-3-nitrobenzylidene)-10-oxododecahydro-1H,5H,8H-dipyrido[2,1-f:3',2',1'-ij][1,6]naphthyridine-11-carbohydrazide (3-nitro-4-hydroxy-benzaldehyde matrine-14-carbohydrazone, **9**)

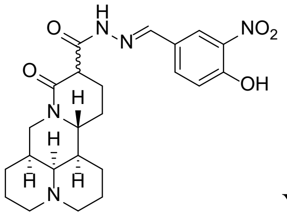

Yellow solid 0.38 g, yield 57%. Mp 177−178 ºC. ^{1}H NMR (400 MHz, DMSO-d_6) δ 11.50 – 11.31 (4s, 1H, NH), 8.15 (d, J = 8.0 Hz, 1H), 8.07 – 7.90 (4 s, 1H, CH=N), 7.84 – 7.78 (m, 1H), 7.15 – 7.05 (m, 1H), 4.22 – 4.14 (m, 1H), 3.88 – 3.76 (m, 1H), 3.10 – 2.84 (m, 1H), 2.87 – 2.81 (m, 3H), 2.31 – 1.80 (m, 7H), 1.62 – 1.17 (m, 10H). HRMS（ESI） calcd for $C_{23}H_{30}N_5O_5$ $[M+H]^+$ 456.2241, found 456.2242.

(41S,7aS,13aR,13bR)-N'-((E)-3-Bromo-4-hydroxybenzylidene)-10-oxododecahydro-1H,5H,8H-dipyrido[2,1-f:3',2',1'-ij][1,6]naphthyridine-11-carbohydrazide (3-bromo-4-hydroxy-benzaldehyde matrine-14-carbohydrazone, **10**)

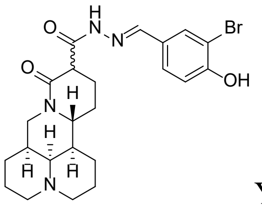

Yellow solid 0.98 g, yield 80%. Mp 170−171 ºC. ^{1}H NMR (400 MHz, DMSO-d_6) δ 11.42 – 11.22 (4 s, 1H, NH), 10.81 (brs, 1H, OH), 8.08 – 7.07 (m,

2H), 7.54 – 7.44 (m, 1H), 7.10 – 6.97 (m, 1H), 4.16 (m, 1H), 3.85 – 3.78 (m, 1H), 3.17 – 3.06 (m, 1H), 3.03 – 2.95 (m, 1H), 2.87 – 2.70 (m, 2H), 2.19 – 1.77 (m, 7H), 1.44 (m, 10H). HRMS（ESI）calcd for $C_{23}H_{30}BrN_4O_3$ $[M+H]^+$ 489.1496, found 489.1504 and 491.1527.

(41S,7aS,13aR,13bR)-N'-((E)-4-Hydroxy-3,5-dimethylbenzylidene)-10-oxododecahydro-1H,5H,8H-dipyrido[2,1-f:3',2',1'-ij][1,6]naphthyridine-11-carbohydrazide (3,5-dmethyl-4- hydroxybenzaldehyde matrine-14-carbohydrazone, **11**)

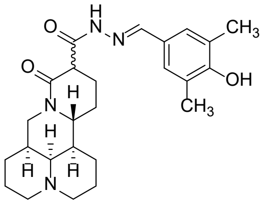

Light yellow solid 0.38 g, yield 54.3%. Mp 168−169 ºC. ^{1}H NMR (400 MHz, DMSO-d_6) δ 13.16 – 11.29 (4 s, 1H, NH), 8.64 (s, 1H, OH), 8.50 – 7.50 (4 s, 1H, CH=N), 7.17 – 6.88 (m, 2H), 4.21 – 4.18 (m, 1H), 3.72 – 3.71 (m, 1H), 3.55 – 3.31 (m, 1H), 2.96 – 2.88 (m, 1H), 2.75 (s, 2H), 2.22 – 1.87 (m, 7H), 1.70 – 1.16 (m, 10H). HRMS（ESI）calcd for $C_{25}H_{35}N_4O_3$ $[M+H]^+$ 439.2704, found 439.2710.

(41S,7aS,13aR,13bR)-N'-((E)-4-Hydroxybenzylidene)-10-oxododecahydro-1H,5H,8H-dipyrido[2,1-f:3',2',1'-ij][1,6]naphthyridine-11-carbohydrazide (4-chlorobenzaldehyde matrine-14-carbohydrazone, **12**)

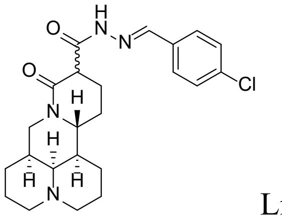

Light yellow solid 0.78 g, yield 60%. Mp 169−170 ºC. ^{1}H NMR (400 MHz, DMSO-d_6) δ 11.55 – 11.41 (s, 1H, NH), 8.22 – 7.95 (4 s, 1H, CH=N), 7.74 – 7.68 (m, 2H), 7.53 – 7.45 (m, 2H), 4.22 – 4.16 (m, 1H), 3.96 – 3.76 (m, 1H), 3.39 – 3.30 (m, 1H), 3.03 – 2.96 (m, 1H), 2.83 – 2.72 (m, 2H), 2.19 – 1.80 (m, 7H), 1.60 – 1.24 (m, 10H). HRMS（ESI）calcd for $C_{23}H_{30}ClN_4O_2$ $[M+H]^+$ 429.2052, found 429.2048.

(41S,7aS,13aR,13bR)-N'-((E)-4-Bromobenzylidene)-10-oxododecahydro-1H,5H,8H-dipyrido[2,1-f:3',2',1'-ij][1,6]naphthyridine-11-carbohydrazide (4-bromobenzaldehyde matrine-14-carbohydrazone, **13**)

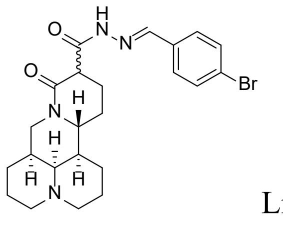

Light yellow solid 0.52 g, yield 69%. Mp 161−162 ºC. ^{1}H NMR (400 MHz, DMSO-d_6) δ 11.56 – 11.41 (4s, 1H, NH), 8.20 – 7.93 (4s, 1H, CH=N), 7.64 – 7.60 (m, 4H), 4.23 – 4.12 (m, 1.3H), 3.84 – 3.82 (m, 1.2H), 3.46 – 2.75 (m, 3.5H), 2.20 – 1.86 (m, 7H), 1.56 – 1.15 (m, 10H). HRMS（ESI）calcd for $C_{23}H_{30}BrN_4O_2$ $[M+H]^+$ 473.1547, found 473.1552 and 475.1535.

(41S,7aS,13aR,13bR)-10-oxo-N'-((E)-4-(Trifluoromethoxy)benzylidene)dodecahydro-1H,5H,8H-dipyrido[2,1-f:3',2',1'-ij][1,6]naphthyridine-11-carbohydrazide (4-trifluoromethoxybenzaldehyde matrine-14-hydrazone, **14**)

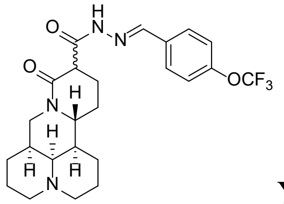

Yellow solid 0.42 g, yield 55%. Mp 176−177 ºC. ^{1}H NMR (400 MHz, DMSO-d_6) δ 11.60 – 11.43 (4s, 1H, NH), 8.25 – 7.97 (4s, 1H, CH=N), 7.84 – 7.76 (m, 2H), 7.46 – 7.37 (m, 2H), 4.21 – 4.11 (m, 1H), 3.94 – 3.76 (m, 1H), 3.56 – 3.14 (m, 1H), 3.04 – 2.91 (m, 1H), 2.89 – 2.73 (m, 2H), 2.21 – 1.20 (m, 17H). HRMS（ESI）calcd for $C_{24}H_{30}F_3N_4O_3$ $[M+H]^+$ 479.2265, found 479.2260.

(41S,7aS,13aR,13bR)-N'-((E)-3-Nitrobenzylidene)-10-oxododecahydro-1H,5H,8H-dipyrido[2,1-f:3',2',1'-ij][1,6]naphthyridine-11-carbohydrazide (m-nitrobenzaldehyde matrine-14-carbohydrazone, **15**)

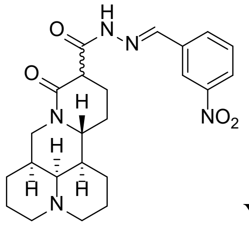

Yellow solid 0.28 g, yield 67%. Mp 184−185 ºC. ^1H NMR (400 MHz, DMSO-d_6) δ 11.78 – 11.59 (4s, 1H, NH), 8.54 – 8.04 (m, 3H), 7.89 (s, 1H), 7.76 – 7.57 (m, 1H), 4.24 – 4.13 (m, 1H), 3.89 – 3.78 (m, 1H), 3.38 (s, 1H), 3.02 – 2.91 (m, 1H), 2.75 (s, 2H), 2.20 – 1.23 (m, 17H). HRMS（ESI）calcd for $C_{23}H_{30}N_5O_4$ $[M+H]^+$ 440.2292, found 440.2300.

(41S,7aS,13aR,13bR)-N'-((E)-1-(4-Nitrophenyl)ethylidene)-10-oxododecahydro-1H,5H,8H-dipyrido[2,1-f:3',2',1'-ij][1,6]naphthyridine-11-carbohydrazide (p-nitroacetophenone matrine-14-carbohydrazone, **16**)

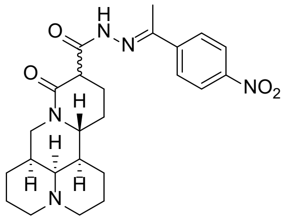

Yellow solid 0.54 g，yield 75%. Mp 168−169 ºC。^1H NMR (400 MHz, DMSO-d_6) δ 10.91 – 10.81 (4s, 1H, NH), 8.27 – 7.86 (m, 4H), 4.30 – 4.05 (m, 1H), 3.82 (s, 1H), 3.64 – 3.50 (m, 0.50H), 3.04 – 2.94 (m, 0.50H), 2.85 – 2.68 (m, 2H), 2.33 – 2.28 (4s, 3H), 2.24 – 1.85 (m, 7 H), 1.68 – 1.23 (m, 10H). HRMS（ESI）calcd for $C_{24}H_{32}N_5O_4$ $[M+H]^+$ 454.2356, found, 454.2355.

(41S,7aS,13aR,13bR)-N'-((E)-3-Methylbenzylidene)-10-oxododecahydro-1H,5H,8H-dipyrido[2,1-f:3',2',1'-ij][1,6]naphthyridine-11-carbohydrazide (3-cyanobenzaldehyde matrine-14-carbohydrazone, **17**)

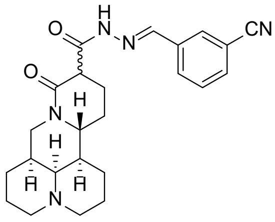

Light yellow solid 0.38 g, yield 58%. Mp 177−178 ºC. ^1H NMR (400 MHz, DMSO-d_6) δ 11.71 – 11.54 (s, 1H, NH), 8.78 – 8.23 (4s, 1H, CH=N), 8.10 – 8.03 (m, 1H), 8.05 – 7.08 (m, 1H), 7.87 (t, J = 8.0 Hz, 1H), 7.75 – 7.50 (m, 1H),

4.26 –4.16 (m, 1.3H), 4.00 – 3.83 (m, 1.2H), 3.03 – 2.95 (m, 1.5H), 2.82 – 2.71 (m, 2H), 2.17 – 1.87 (m, 7H) , 1.68 – 1.34 (m, 10H). HRMS（ESI）calcd for $C_{24}H_{30}N_5O_2$ $[M+H]^+$ 420.2394, found 420.2402.

(41S,7aS,13aR,13bR)-N'-((E)-4-Methylbenzylidene)-10-oxododecahydro-1H,5H,8H-dipyrido[2,1-f:3',2',1'-ij][1,6]naphthyridine-11-carbohydrazide (4-cyano-benzaldehyde matrine-14-carbohydrazone, **18**)

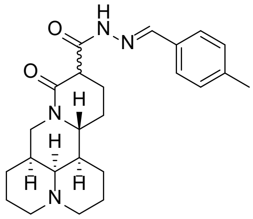

Yellow solid 0.18 g, yield 64%. Mp 180−181 ºC. ^1H NMR (400 MHz, DMSO-d_6) δ 11.76 – 11.62 (4s, 1H, NH), 8.27 – 8.01 (4s, 1H, CH=N), 7.91 – 7.82 (m, 4H), 4.26 – 4.12 (m, 1H), 4.01 – 3.75 (m, 1H), 3.35 (s, 1H), 3.18 – 2.02 (m, 1H), 2.76 (s, 2H), 2.18 – 1.12 (m, 17H). HRMS（ESI）calcd for $C_{24}H_{30}N_5O_3$ $[M+H]^+$ 420.2394, found 420.2402.

(41S,7aS,13aR,13bR)-N'-((E)-Naphthalen-1-ylmethylene)-10-oxododecahydro-1H,5H,8H-dipyrido[2,1-f:3',2',1'-ij][1,6]naphthyridine-11-carbohydrazide (1-naphthaldehyde matrine-14-carbohydrazone, **19**)

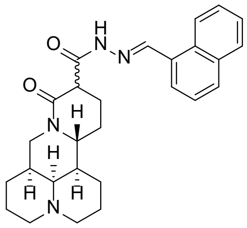

Yellow solid 0.46 g, yield 65%. Mp 148−149 °C. ^1H NMR (400 MHz, DMSO-d_6) δ 11.58 – 11.36 (m, 1H, NH), 8.37 – 8.11 (m, 2H), 8.06 – 7.85 (m, 4H), 7.56 – 7.46 (m, 2H), 4.31 – 4.16 (m, 1H), 3.96 – 3.81 (m, 1H), 3.33 – 2.71 (m, 4H), 2.25 – 1.34 (m, 17H). HRMS（ESI）calcd for $C_{27}H_{33}N_4O_2$ $[M+H]^+$ 445.2598, found 445.2603.

(41S,7aS,13aR,13bR)-N'-((E)-Butylidene)-10-oxododecahydro-1H,5H,8H-dipyrido[2,1-f:3',2',1'-ij][1,6]naphthyridine-11-carbohydrazide (n-butyraldehyde

matrine-14-carbohydrazone, **20**)

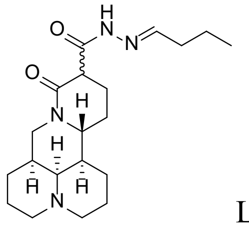

Light yellow solid 0.39 g, yield 68%. Mp 144-145 ºC. ^{1}H NMR (400 MHz, DMSO-d_6) δ 11.03 – 10.88 (s, 1H, NH), 7.90 – 7.02 (m, 1H, CH=N), 4.20 – 4.12 (m, 1H), 3.82 – 3.69 (m, 2H), 3.24 – 2.91 (m, 1H), 2.75 (s, 2H), 2.19 – 1.47 (m, 21H), 0.92 – 0.86 (m, 3H). HRMS（ESI）calcd for $C_{20}H_{33}N_4O_2$ $[M+H]^+$ 361.2598, found 361.2604.

(41S,7aS,13aR,13bR)-N'-((E)-3,3-Dimethylbutan-2-ylidene)-10-oxododecahydro-1H, 5H,8H-dipyrido[2,1-f:3',2',1'-ij][1,6]naphthyridine-11-carbohydrazide (t-butylethylketone matrine-14-carbohydrazone, **21**)

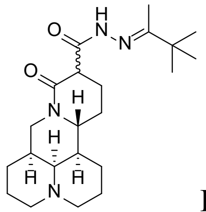

Light yellow solid 0.39 g，yield 67%. Mp 136−137 ºC. ^{1}H NMR (400 MHz, DMSO-d_6) δ 10.16 – 9.97 (4s, 1H, NH), 4.20 – 4.13 (m, 1.27H), 3.76 – 3.71 (m, 1.13H), 3.45 – 3.34 (m, 1.27H), 2.99 – 2.92 (m, 1.13H), 2.77 – 2.71 (m, 2.1H), 2.16 – 2.01 (m, 2H), 1.92 – 1.78 (m, 7H), 1.62 – 1.35 (m, 10H), 1.11 – 1.04 (m, 9H). HRMS（ESI）calcd for $C_{22}H_{36}N_4O_2$ $[M+H]^+$ 389.2911, found 389.2918.

(41S,7aS,13aR,13bR)-N'-((E)-Octylidene)-10-oxododecahydro-1H,5H,8H-dipyrido[2,1-f:3',2',1'-ij][1,6]naphthyridine-11-carbohydrazide (n-octanal matrine-14-carbohydrazone, **22**)

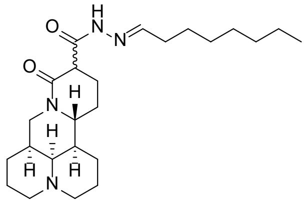

Light yellow solid 0.50 g, yield 75%. Mp 121−122 ºC. ^{1}H NMR (400 MHz, DMSO-d_6) δ 11.13 – 10.77 (m, 1H, NH), 7.48 – 7.12 (m, 1H,

CH=N), 4.17 – 2.91 (m, 4H), 2.75 – 2.74 (m, 2H), 2.30 – 1.77 (m, 7H), 1.58 – 1.16 (m, 22H), 0.83 (s, 3H). HRMS（ESI）calcd for $C_{24}H_{41}N_4O_2$[M+H]$^+$ 417.3224, found 417.3228.

(41S,7aS,13aR,13bR)-N'-((E)-Cyclohexylmethylene)-10-oxododecahydro-1H,5H,8H-dipyrido[2,1-f:3',2',1'-ij][1,6]naphthyridine-11-carbohydrazide (cyclohexyl formaldehyde matrine-14-carbohydrazone, **23**)

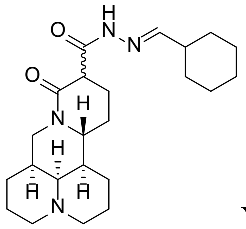

Yellow solid 0.35 g, yield 55%. Mp 142−143 ºC. ^{1}H NMR (400 MHz, DMSO-d_6) δ 10.97 – 10.79 (4s, 1H, NH), 7.65 – 7.35 (m, 1H, CH=N), 4.20 – 4.09 (m, 1H), 3.80 – 3.69 (m, 2H), 2.99 – 2.91 (m, 1H), 2.77 – 2.70 (m, 2H), 2.17 – 2.07 (m, 3H), 1.92 – 1.51 (m, 17H), 1.35 – 1.14 (m, 9H). HRMS（ESI）calcd for $C_{23}H_{37}N_4O_2$ [M+H]$^+$ 401.2918, found 401.2915.

(41S,7aS,13aR,13bR)-10-oxo-N'-((E)-Pyridin-3-ylmethylene)dodecahydro-1H,5H,8H-dipyrido[2,1-f:3',2',1'-ij][1,6]naphthyridine-11-carbohydrazide (3-pyridineformaldehyde matrine-14-carbohydrazone, **24**)

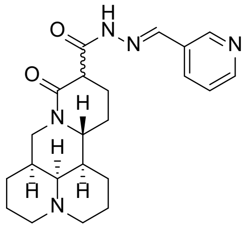

Brown solid 0.40 g, yield 64%. Mp 183−184 °C. ^{1}H NMR (400 MHz, DMSO-d_6) δ 11.66 – 11.44 (m, 1H, NH), 8.84 – 8.77 (m, 1H), 8.60 – 8.24 (m, 1H), 8.26 – 7.99 (m, 2H), 7.49 – 7.44 (m, 1H), 4.24 – 4.10 (m, 1H), 3.82 (s, 1H), 3.17 – 2.97 (m, 2H), 2.78 – 2.71 (m, 2H), 2.23 – 1.20 (m, 17H). HRMS（ESI）calcd for $C_{22}H_{30}N_5O_2$ [M+H]$^+$ 396.2394, found 396.2398.

(41S,7aS,13aR,13bR)-N'-((E)-(5-Methylthiophen-2-yl)methylene)-10-oxododecahydro-1H,5H,8H-dipyrido[2,1-f:3',2',1'-ij][1,6]naphthyridine-11-carbohydrazide

(5-methylthiopheneformaldehyde matrine-14-carbohydrazone, **25**)

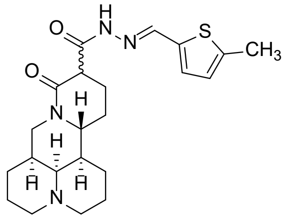

Light yellow solid 0.48 g, yield 73%. Mp 193−194 ºC. ^{1}H NMR (400 MHz, DMSO-d_6) δ 11.38 – 11.19 (m, 1H, NH), 8.71 – 7.85 (m, 1H), 7.48 – 7.15 (m, 1H), 6.93 – 6.77 (m, 1H), 4.22 – 3.57 (m, 2.5H), 3.28 – 2.71 (m, 3.5H), 2.55 – 2.42 (m, 3H), 2.18 –1.79 (m, 7H), 1.63 – 1.36 (m, 10H). HRMS（ESI）calcd for $C_{22}H_{31}N_4O_2S$ $[M+H]^+$ 415.2162, found 415.2162.

4-((1R,3aS,3a1S,10aR)-2-Benzyldecahydro-1H,4H-pyrido[3,2,1-ij][1,6]naphthyridin-1-yl)-N'-((E)-2,4-dimethoxybenzylidene)butanehydrazide

(2,4-dimethoxylbenzaldehyde N-benzyl-11-butanehydrazone of matrine derivative, **26**)

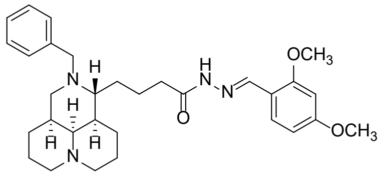

Yellow solid, 0.39 g, yield 66%, Mp 139−141 °C. ^{1}H NMR (400 MHz, DMSO-d_6) δ 11.45 – 11.03 (m, 1H, NH), 8.47 – 8.40 − 8.19 (m, 1H, CH=N), 7.70 – 7.29 (m, 6H), 6.61 – 6.50 (m, 2H), 4.22 – 4.00 (m, 1H), 3.83 – 3.79 (4s, 6H), 3.25 – 2.57 (m, 7H), 2.33 – 1.23 (m, 18H). HRMS (ESI) calcd for $C_{31}H_{43}N_4O_3$ $[M+H]^+$ 519.3330, found 519.3334.

4-((1R,3aS,3a1S,10aR)-2-Benzyldecahydro-1H,4H-pyrido[3,2,1-ij][1,6]naphthyridin-1-yl)-N'-((E)-3,4-dimethoxybenzylidene)butanehydrazide

(3,4-dimethoxylbenzaldehyde *N*-benzylmatrine-11-butanehydrazone, **27**)

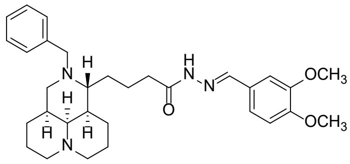

Light yellow solid 0.45 g, yield 63%，Mp 147−148 °C. ^{1}H NMR (400 MHz, DMSO-d_6) δ 11.55 –11.11 (m, 1H, NH), 8.19 – 7.89 (4s, 1H,

CH=N), 7.64 – 6.92 (m, 8H), 4.19 – 3.91 (m, 1H), 3.82 – 3.76 (m, 6H), 3.25 – 3.16 (m, 2H), 2.95 – 2.51 (m, 4H), 2.34 – 1.23 (m, 19H). HRMS（ESI）calcd for $C_{31}H_{43}N_4O_3$ $[M+H]^+$ 519.3330, found 519.3338.

4-((1R,3aS,3a1S,10aR)-2-Benzyldecahydro-1H,4H-pyrido[3,2,1-ij][1,6]naphthyridin-1-yl)-N'-((E)-4-hydroxy-3,5-dimethylbenzylidene)butanehydrazide (3,5-dimethoxyl-4-hydroxybenzaldehyde *N*-benzylmatrine-11-butanehydrazone, **28**)

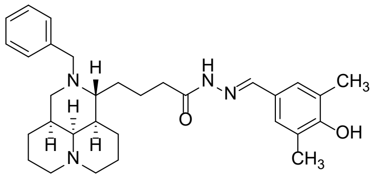

Light yellow solid 0.40 g, yield 62%, Mp 149−150 °C. ^{1}H NMR (400 MHz, DMSO-d_6) δ 11.45 – 10.97 (4s, 1H, NH), 8.72 – 8.68 (2s, 1H, OH), 8.10 – 7.83 (m, 1H, CH=N), 7.65 – 7.20 (m, 7H), 4.19 – 3.90 (m, 1H), 3.22 – 2.60 (m, 7H), 2.33 – 1.13 (m, 24H). HRMS（ESI）calcd for $C_{31}H_{42}N_4O_2$ $[M+H]^+$ 503.3381, found 503.3387.

4-((1R,3aS,3a1S,10aR)-2-Benzyldecahydro-1H,4H-pyrido[3,2,1-ij][1,6]naphthyridin-1-yl)-N'-((E)-2-hydroxybenzylidene)butanehydrazide (salicylaldehyde *N*-benzylmatrine-11-butanehydrazone, **29**)

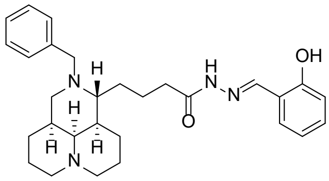

Light yellow solid 0.28 g, yield 43%, Mp 146−147 °C. ^{1}H NMR (400 MHz, DMSO-d_6) δ 11.93 – 11.16 (m, 2H, NH+OH), 8.45 – 8.25 (4s, 1H, CH=N), 7.63 – 7.37 (m, 1H), 7.34 – 7.22 (m, 4H), 6.92 – 6.81 (m, 2H), 4.19 – 3.92 (m, 1H), 3.60 – 3.00 (m, 3H), 3.05 – 2.59 (m, 4H), 2.41 – 1.34 (m, 18H). HRMS（ESI）calcd for $C_{29}H_{39}N_4O_2$ $[M+H]^+$ 475.3068, found 475.3073.

4-((1R,3aS,3a1S,10aR)-2-Benzyldecahydro-1H,4H-pyrido[3,2,1-ij][1,6]naphthyridin-1-yl)-N'-((E)-3-hydroxybenzylidene)butanehydrazide (3-hydroxybenzaldehyde *N*-benzylmatrine-11-butanehydrazone, **30**)

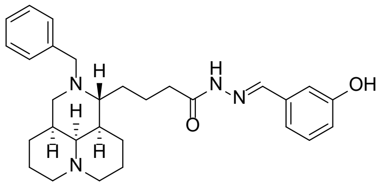

Light yellow solid 0.32 g, yield 49%, Mp 129−130 °C. ^{1}H NMR (400 MHz, DMSO-d_6) δ 11.62 – 11.16 (4s, 1H, NH), 9.65 – 9.63 (2s, 1H, OH), 8.19 – 7.88 (4s, 1H, CH=N), 7.65 – 7.01 (m, 8H), 6.82 – 6.80 (m, 1H), 4.19 – 3.91 (m, 1H), 3.37 – 2.62 (m, 7H), 2.33 – 1.23 (m, 18H). HRMS（ESI）calcd for $C_{29}H_{39}N_4O_2$ $[M+H]^+$ 475.3068, found 475.3075.

4-((1R,3aS,3a1S,10aR)-2-Benzyldecahydro-1H,4H-pyrido[3,2,1-ij][1,6]naphthyridin-1-yl)-N'-((E)-4-bromo-2-hydroxybenzylidene)butanehydrazide (2-hydroxy-4-bromobenzaldehyde *N*-benzylmatrine-11-butanehydrazone, **31**)

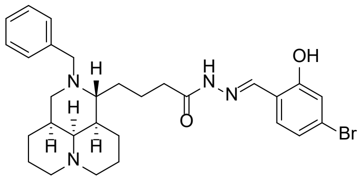

Light yellow solid 0.40, yield 51%, Mp 126−127 °C. ^{1}H NMR (400 MHz, DMSO-d_6) δ 11.78 – 10.44 (4s, 2H, NH+OH), 8.35 – 8.21 (2s, 1H, CH=N), 7.76 – 7.72 (m, 1H), 7.41 - 7.32 (m, 6H), 6.88 (d, J = 8.0 Hz, 1H), 4.22 – 3.98 (m, 1H), 3.31 – 2.60 (m, 7H), 2.28 – 1.23 (m, 18H). HRMS（ESI）calcd for $C_{29}H_{38}BrN_4O_2$ $[M+H]^+$ 553.2173, found 553.2188 and 555.2188.

4-((1R,3aS,3a1S,10aR)-2-Benzyldecahydro-1H,4H-pyrido[3,2,1-ij][1,6]naphthyridin-1-yl)-N'-((E)-4-chlorobenzylidene)butanehydrazide (4-chlorobenzaldehyde *N*-benzylmatrine-11-butanehydrazone, **32**)

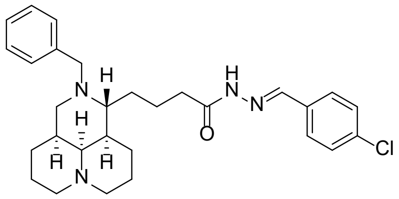

Light yellow solid 0.45 g, yield 65%. Mp 131−132 °C。^{1}H NMR (400 MHz, DMSO-d_6) δ 11.71 – 11.30 (m, 1H, NH), 8.24 – 7.94 (m, 1H, CH=N), 7.69 – 7.65 (m, 2H), 7.56 – 6.73 (m, 7H), 4.23 – 3.84 (m, 1H), 3.22 – 2.65 (m, 7H), 2.33 – 1.16 (m, 18H). HRMS（ESI）calcd for $C_{29}H_{38}ClN_4O$ $[M+H]^+$ 493.2729, found 493.2736.

4-((1R,3aS,3a1S,10aR)-2-Benzyldecahydro-1H,4H-pyrido[3,2,1-ij][1,6]naphthyridin-1-yl)-N'-((E)-3,4-dichlorobenzylidene)butanehydrazide (3,4-dichlorobenzaldehyde *N*-benzylmatrine-11-butanehydrazone, **33**)

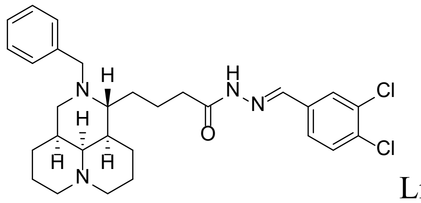

Light yellow solid 0.40 g, yield 55%. Mp 120−121 °C. ^{1}H NMR (400 MHz, DMSO-d_6) δ 11.80 – 11.38 m, 1H, NH), 8.14 (s, 0.3H, CH=N), 7.93 – 7.88 (m, 1.3H, containing CH=N), 7.68 – 7.62 (m, 2H), 7.47 – 7.23 (m, 5H), 4.13 – 4.02 (m, 1H), 3.18 – 3.07 (m, 3H), 2.87 – 2.63 (m, 4H). 2.32 – 1.23 (m, 18H). HRMS（ESI）calcd for $C_{29}H_{37}Cl_2N_4O$ $[M+H]^+$ 527.2339, found 527.2346.

4-((1R,3aS,3a1S,10aR)-2-Benzyldecahydro-1H,4H-pyrido[3,2,1-ij][1,6]naphthyridin-1-yl)-N'-((E)-3-bromobenzylidene)butanehydrazide (3-bromobenzaldehyde *N*-benzylmatrine-11-butanehydrazone, **34**)

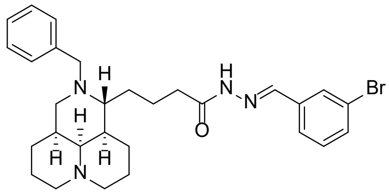

Brown solid 0.28 g, yield 57%. Mp 140−141 °C. ^{1}H NMR (400 MHz, DMSO-d_6) δ 11.67 – 11.25 (4s, 1H, NH), 8.41 – 8.04 (m, 1H, N=CH), 7.95 – 7.92 (m, 1H), 7.87 – 7.80 (m, 1H), 7.66 – 7.56 (m, 1H), 7.48 – 7.22 (m, 6H), 4.24 – 4.15 (m, 1H), 3.36 – 2.26 (m, 7H), 2.33 – 1.23 (m, 18H). HRMS（ESI）calcd for $C_{29}H_{38}BrN_4O$ $[M+H]^+$ 537.2224, found 537.2227 and 539.2224.

4-((1R,3aS,3a1S,10aR)-2-Benzyldecahydro-1H,4H-pyrido[3,2,1-ij][1,6]naphthyridin-1-yl)-N'-((E)-3-cyanobenzylidene)butanehydrazide (3-cyanobenzaldehyde *N*-benzylmatrine-11-butanehydrazone, **35**)

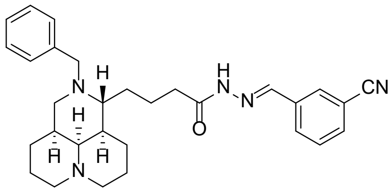

Light yellow solid 0.31 g, yield 67%. Mp 134−135 °C. ^{1}H NMR (400 MHz, DMSO-d_6) δ 11.81 – 11.42 (2s, 1H, NH), 8.23 (s, 0.3H, CH=N), 8.07 (s, 1H), 8.02 – 8.79 (m, 1.4H, containing CH=N), 7.88 – 7.82 (m, 1H), 7.66 – 7.57 (m, 1H), 7.50 – 7.17 (m, 5H), 4.22 – 3.96 (m, 1H), 3.27 – 3.09 (m, 2H), 2.87 – 2.66 (m, 5H), 2.33 – 1.23 (m, 18H). HRMS（ESI）calcd for $C_{30}H_{38}N_5O$ $[M+H]^+$ 484.3071, found 484.3076.

4-((1R,3aS,3a1S,10aR)-2-Benzyldecahydro-1H,4H-pyrido[3,2,1-ij][1,6]naphthyridin-1-yl)-N'-((E)-2-(trifluoromethyl)benzylidene)butanehydrazide (2-trifluoro methyl benzaldehyde *N*-benzylmatrine-11-butanehydrazone, **36**)

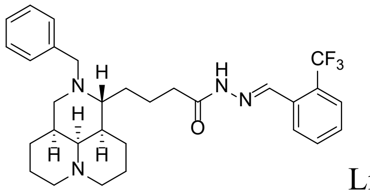

Light yellow solid 0.44 g, yield 60%. Mp 144−145 °C. ^{1}H NMR (400 MHz, DMSO-d_6) δ 11.88 (s, 0.4H, NH), 11.51 (s, 0.6H, NH), 8.57 (s, 0.4H, CH=N), 8.33 (s, 0.6H, CH=N), 8.13 (d, J = 8.0 Hz, 1H), 7.84 – 7.52 (m, 3H), 7.37 – 7.24 (m, 5H), 4.20 – 3.97 (m, 1H), 2.90 – 2.82 (m, 7H), 2.30 (s, 2H), 1.93 – 1.23 (m, 16H). HRMS（ESI）calcd for $C_{30}H_{38}F_3N_4O$ $[M+H]^+$ 527.2992, found 527.3001.

4-((1R,3aS,3a1S,10aR)-2-Benzyldecahydro-1H,4H-pyrido[3,2,1-ij][1,6]naphthyridin-1-yl)-N'-((E)-3-nitrobenzylidene)butanehydrazide (11-m-nitrobenzaldehyde *N*-benzylmatrine-11-butanehydrazone, **37**)

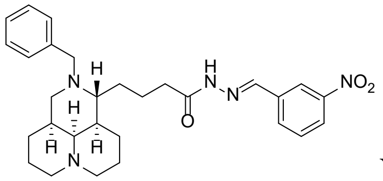

Yellow solid 0.41 g, yield 57%. Mp 187−188 °C.^{1}H NMR (400 MHz, DMSO-d_6) δ 11.88 – 11.46 (m, 1H, NH), 8.48 – 8.41(2s, 1H), 8.41 – 8.08 (m, 3H, containing CH=N), 7.74 – 7.15 (m, 6H), 4.22 – 3.98 (m, 1H), 3.28 –

3.20 (m, 2H), 3.09 – 2.64 (m, 5H), 2.33 – 1.26 (m, 18H). HRMS（ESI）calcd for $C_{29}H_{37}N_5O_3$ $[M+H]^+$ 504.2969, found 504.2975.

4-((1R,3aS,3a1S,10aR)-2-Benzyldecahydro-1H,4H-pyrido[3,2,1-ij][1,6]naphthyridin-1-yl)-N'-((E)-naphthalen-1-ylmethylene)butanehydrazide (1-naphthaldehyde *N*-benzylmatrine-11-butanehydrazone, **38**)

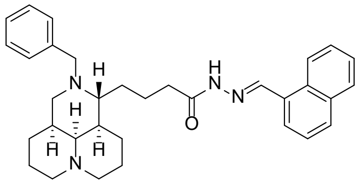

Yellow solid 0.52 g, yield 73%, Mp 125−126 °C. ^{1}H NMR (400 MHz, DMSO-d_6) δ 11.69 – 11.35 (2s, 1H, NH), 8.50 – 8.15 (3s, 1H, CH=N), 8.04 – 7.91 (m, 5H), 7.55 – 7.20 (m, 7H), 4.14 – 4.01 (m, 1H), 3.38 (s, 2H), 2.89 – 2.51 (m, 5H), 2.30 – 1.22 (m, 18H). HRMS（ESI）calcd for $C_{33}H_{41}N_4O$ $[M+H]^+$ 509.3275,found 509.3285.

4-((1R,3aS,3a1S,10aR)-2-Benzyldecahydro-1H,4H-pyrido[3,2,1-ij][1,6]naphthyridin-1-yl)-N'-((E)-(6-hydroxynaphthalen-1-yl)methylene)butanehydrazide (6-hydroxy-1-naphthylformaldehyde *N*-benzylmatrine-11-butanehydrazone, **39**)

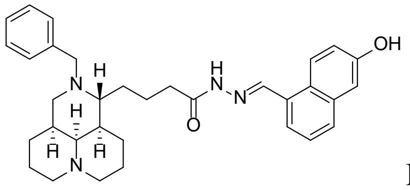

Light yellow solid 0.40 g, yield 55%, Mp 119−120 °C. ^{1}H NMR (400 MHz, DMSO-d_6) δ 11.70 – 11.21 (m, 1H, NH), 10.08 – 10.04 (2s, 1H, OH), 8.38 – 8.08 (m, 1H, CN=N), 8.00 – 7.67 (m, 5H), 7.45 – 7.12 (m, 6H), 4.15 – 3.91 (m, 1H), 3.38 – 2.67 (m, 7H), 2.34 – 1.16 (m, 18H). HRMS（ESI）calcd for $C_{33}H_{40}N_4O_2$ $[M+H]^+$ 525.3224, found 525.3233.

4-((1R,3aS,3a1S,10aR)-2-Benzyldecahydro-1H,4H-pyrido[3,2,1-ij][1,6]naphthyridin-1-yl)-N'-((E)-thiophen-3-ylmethylene)butanehydrazide (3-thiophene formaldehyde *N*-benzylmatrine-11-butanehydrazone, **40**)

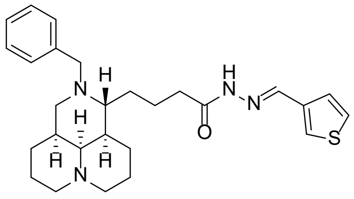

Light yellow solid 0.40 g, yield 62%. Mp 178−179 °C. ^{1}H NMR (400 MHz, DMSO-d_6) δ 11.68 – 9.89 (m, 1H, NH), 8.49 (s, 0.5H), 8.16 (s, 0.5H), 7.63 – 7.10 (m, 8H), 4.16 – 3.93 (m, 1H), 4.35 – 3.14 – 1.70 (m, 7H), 2.31 – 1.23 (m, 18H). HRMS（ESI）calcd for $C_{27}H_{36}N_4OS$ $[M+H]^+$ 465.2683, found 465.2688.

4-((1R,3aS,3a1S,10aR)-2-Benzyldecahydro-1H,4H-pyrido[3,2,1-ij][1,6]naphthyridin-1-yl)-N'-((E)-pyridin-3-ylmethylene)butanehydrazide (3-pyridineformaldehyde *N*-benzylmatrine-11-butanehydrazone, **41**)

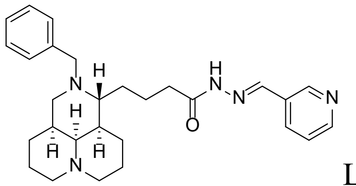

Light yellow solid 0.48，yield75%，Mp 170−171 °C。^{1}H NMR (400 MHz, DMSO-d_6) δ 11.76 – 11.42 (2s, 1H, NH), 9.07 (s, 0.1H, CH=N), 8.79 –8.74 (2s, 1H), 8.58 – 8.55 (m, 1H), 8.27 (s, 0.45H, CH=N), 8.07 – 8.03 (m, 1H), 8.01 (s, 0.45H, CH=N), 7.50 – 7.27 (m, 6H), 4.14 (s, 1H), 3.34 – 2.67 (m, 7H), 2.29 (s, 2H), 1.93 – 1.23 (m, 16H). HRMS（ESI）calcd for $C_{28}H_{38}N_5O$ $[M+H]^+$ 460.3071, found 460.3075.

4-((1R,3aS,3a1S,10aR)-2-Benzyldecahydro-1H,4H-pyrido[3,2,1-ij][1,6]naphthyridin-1-yl)-N'-((E)-(4-bromo-1H-indol-3-yl)methylene)butanehydrazide (4-bromine-3-indoleformaldehyde *N*-benzylmatrine-11-butanehydrazone, **42**)

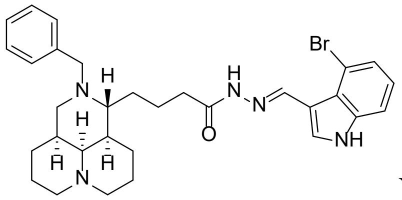

Yellow solid 0.48, yield 65%, Mp 143−144 °C. ^{1}H NMR (400 MHz, DMSO-d_6) δ 11.79 – 11.75 (2s, 1H, indole-NH), 11.40 – 10.97 (brs, 1H, NH), 8.35 (brs, 1H, CH=N), 8.27 (s, 0.5H), 8.13 (s, 0.5H), 7.83 – 7.80 (m, 1H), 7.50 – 7.24 (m, 7H), 4.03 – 3.88 (m, 1H), 3.50 – 3.40 (m, 2H), 2.89 – 2.68 (m, 5H),

2.32 – 1.23 (m, 18H). HRMS（ESI）calcd for $C_{31}H_{37}BrN_5O$ $[M+H]^+$ 576.2332, found 576.2366 and 578.2367.

4-((1R,3aS,3a1S,10aR)-2-Benzyldecahydro-1H,4H-pyrido[3,2,1-ij][1,6]naphthyridin-1-yl)-N'-((E)-octylidene)butanehydrazide (n-octanal *N*-benzylmatrine-11-butanehydrazone, **43**)

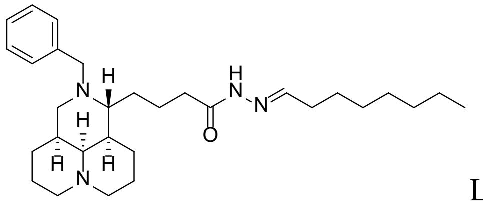

Light yellow oil 0.34 g, yield 72%. 1H NMR (400 MHz, DMSO) δ 11.18 – 10.77 (3s, 1H, NH), 8.00 – 7.65 (3s, 1H, CH=N), 7.49 – 7.32 (m, 5H), 4.17 – 3.82 (m, 1H), 3.17 – 2.73 (m, 6H), 2.27 – 1.25 (m, 31H), 0.85 (t, J = 8.0 Hz, 3H). HRMS（ESI）calcd for $C_{30}H_{49}N_4O$ $[M+H]^+$ 481.3906, found 481.3910.

4-((1R,3aS,3a1S,10aR)-2-Benzyldecahydro-1H,4H-pyrido[3,2,1-ij][1,6]naphthyridin-1-yl)-N'-((E)-2,2-dimethylpropylidene)butanehydrazide (^{t}butylformaldehyde *N*-benzylmatrine-11-butanehydrazone, **44**)

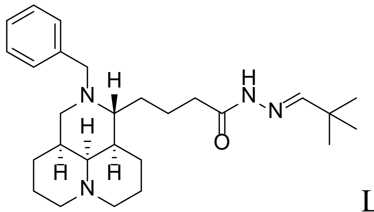

Light yellow oil 0.19 g, yield 61%. ^{1}H NMR (400 MHz, DMSO-d_6) δ 11.20 – 10.72 (s, 1H, NH), 7.66 – 7.43 (2s, 1H, CH=N), 7.41 – 7.19 (m, 5H), 4.19 – 4.02 (m, 1H), 3.24 – 3.16 (m, 2H), 3.03 – 2.68 (m, 5H), 2.33 – 1.23 (m, 18H), 1.11 – 1.01 (3s, 9H). HRMS（ESI）calcd for $C_{27}H_{43}N_4O$ $[M+H]^+$ 439.3431, found 439.3437.

4-((1R,3aS,3a1S,10aR)-2-Benzyldecahydro-1H,4H-pyrido[3,2,1-ij][1,6]naphthyridin-1-yl)-N'-((E)-cyclohexylmethylene)butanehydrazide (cyclohexyl formaldehyde cyclohexyl formaldehyde, **45**)

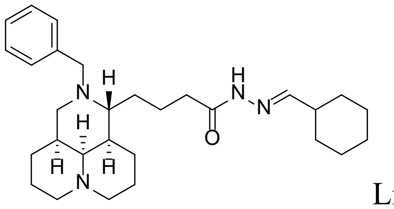

Light yellow 0.35 g, yield 64%, Mp 108−109 °C. ^{1}H NMR (400 MHz, DMSO-d_6) δ 9.77 (s, 1H, NH), 7.96 – 7.50 (m, 1H, CH=N), 7.47 – 7.23 (m, 5H), 4.17 – 4.02 (m, 1H), 3.24 – 2.65 (m, 6H), 2.37 – 2.23 (m, 5H), 1.97 – 1.23 (m, 25H). HRMS（ESI）calcd for $C_{29}H_{45}N_4O$ $[M+H]^+$ 465.3588, found 465.3592.

2. NMR spectra of intermediates A–D and target compounds 1–45

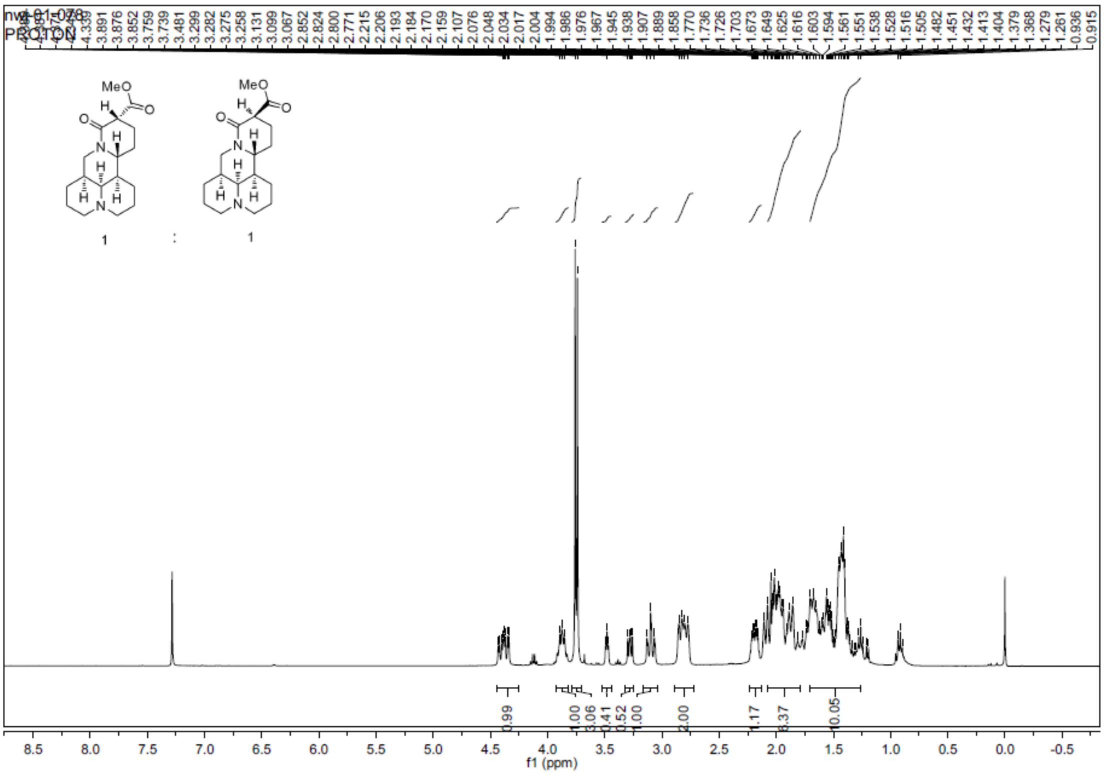

^{1}H NMR of A

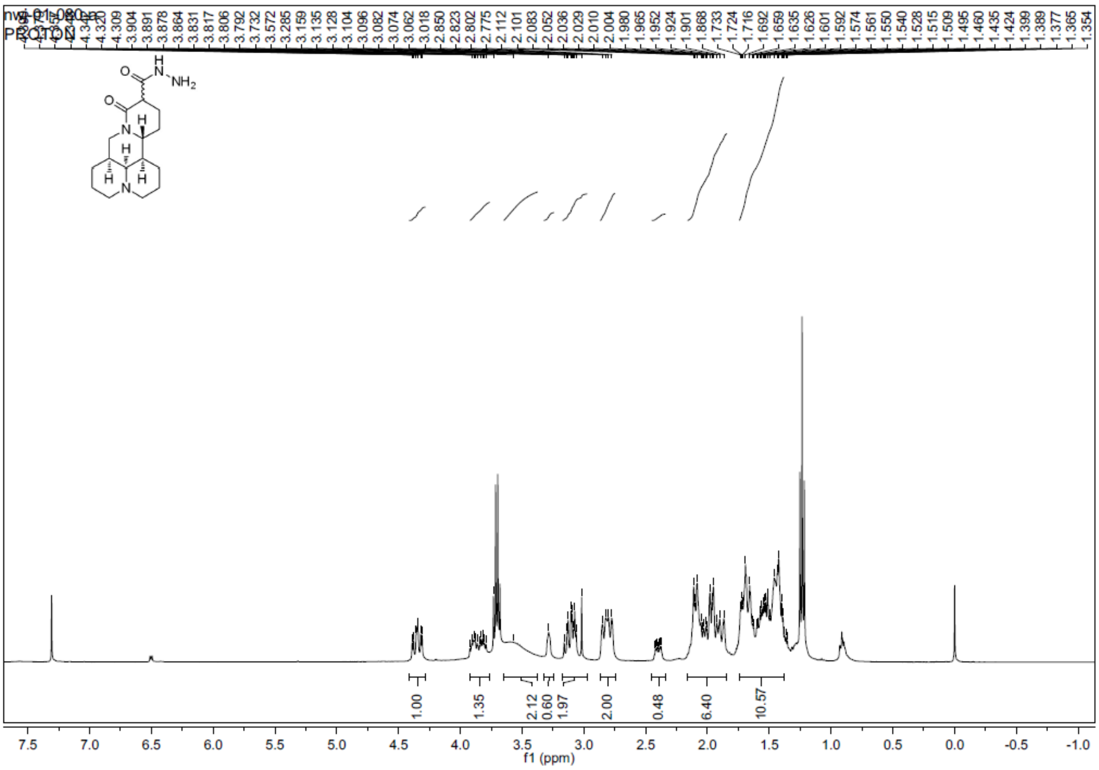

^{1}H NMR of B

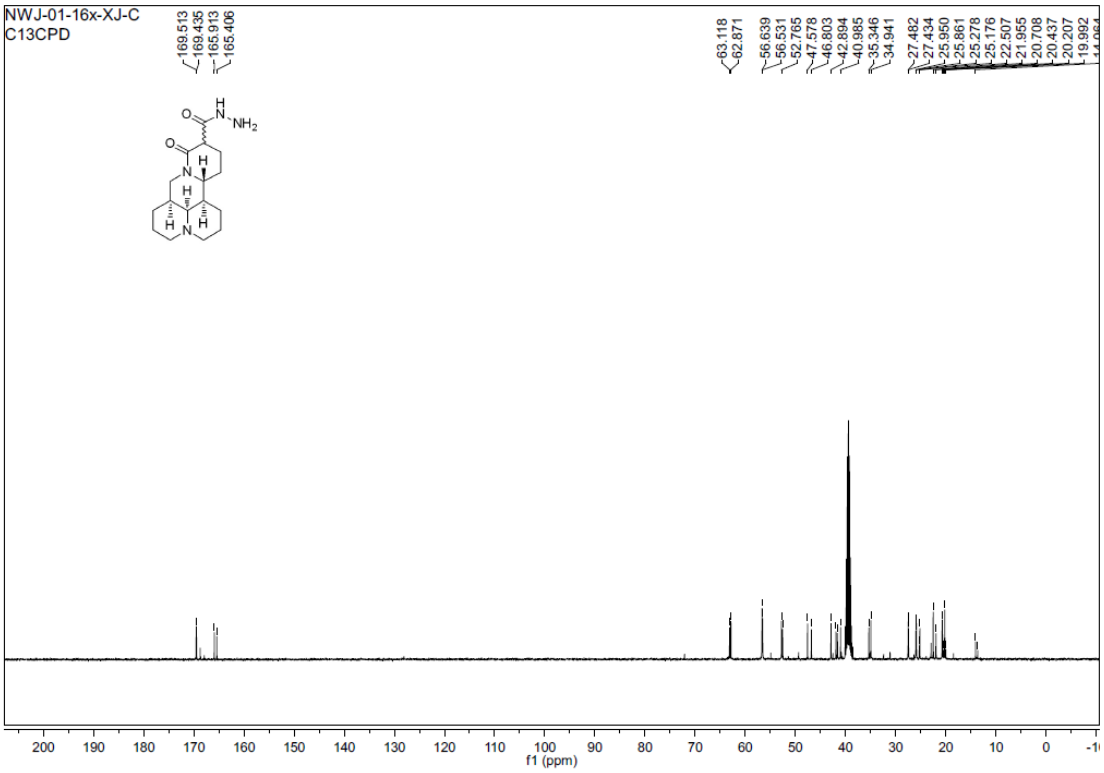

^{13}C NMR of B

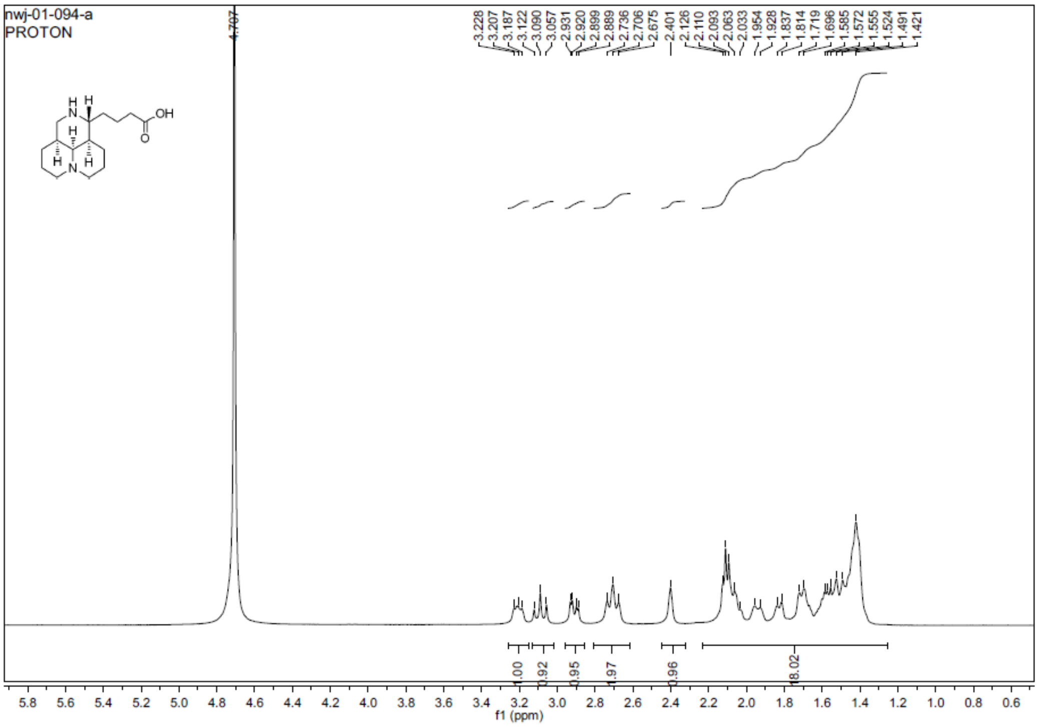

^{1}H NMR of C

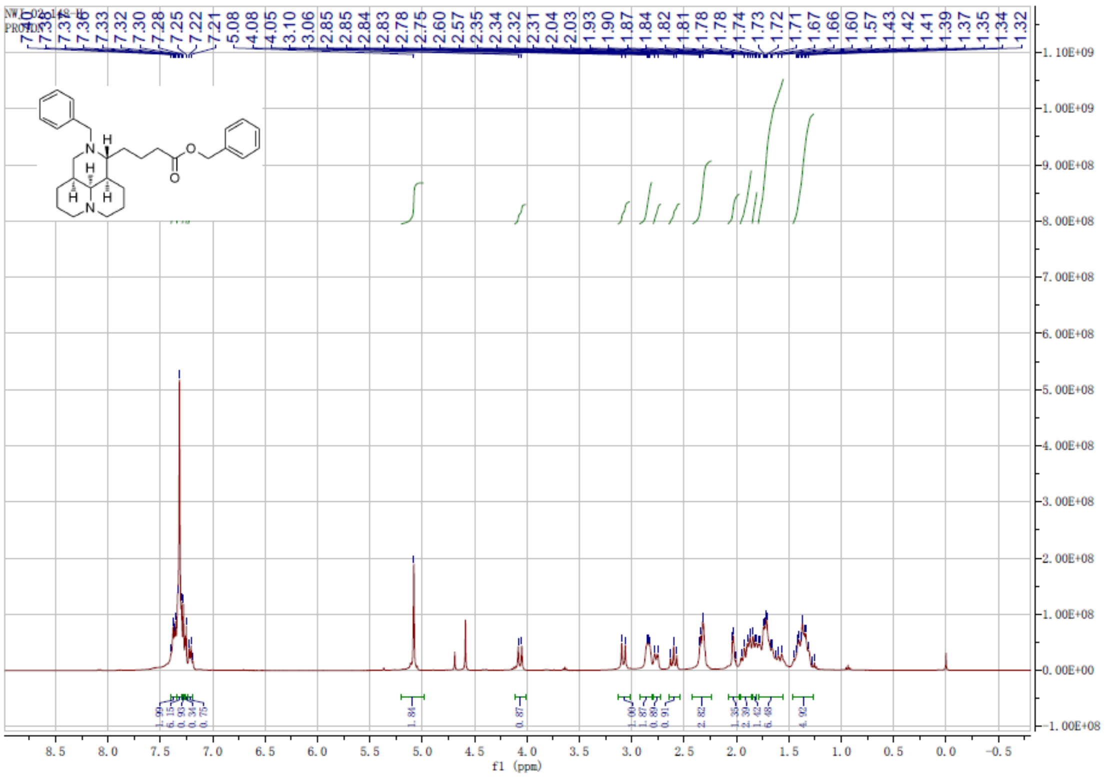

^{1}H NMR of D

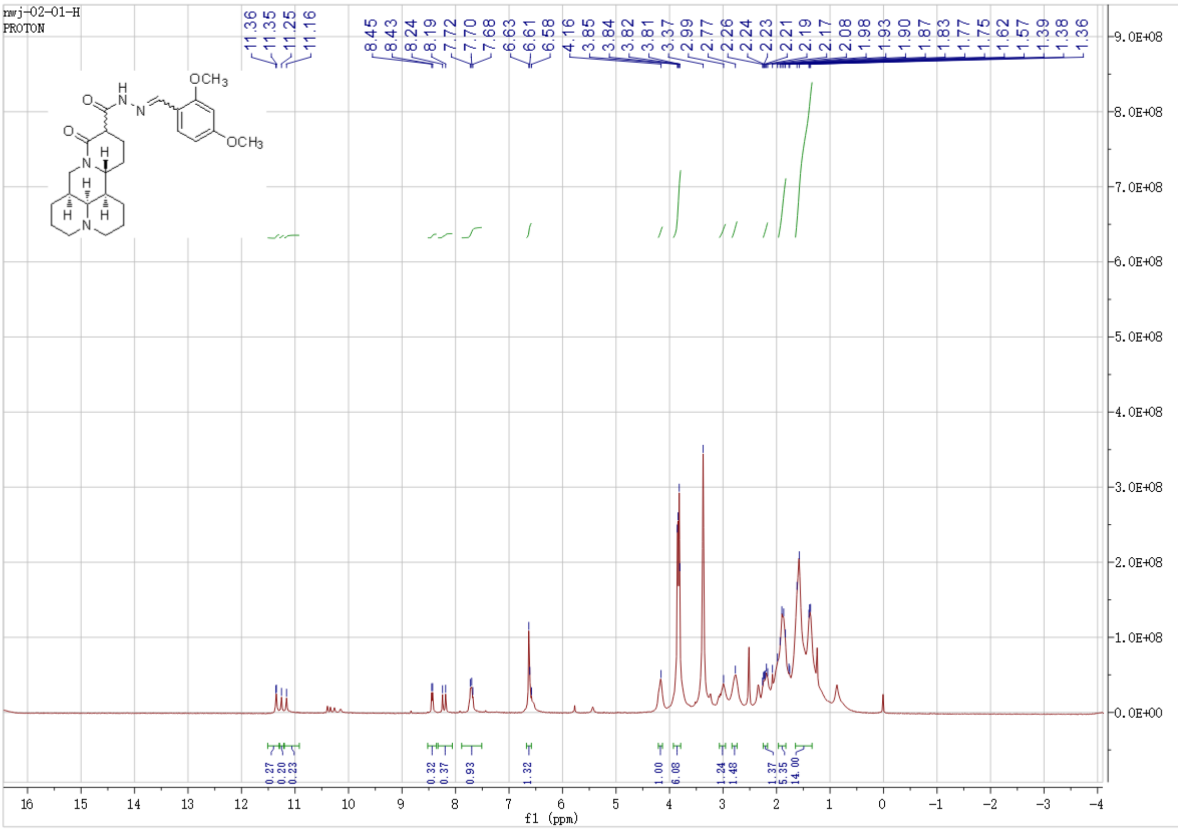

^{1}H NMR of 1

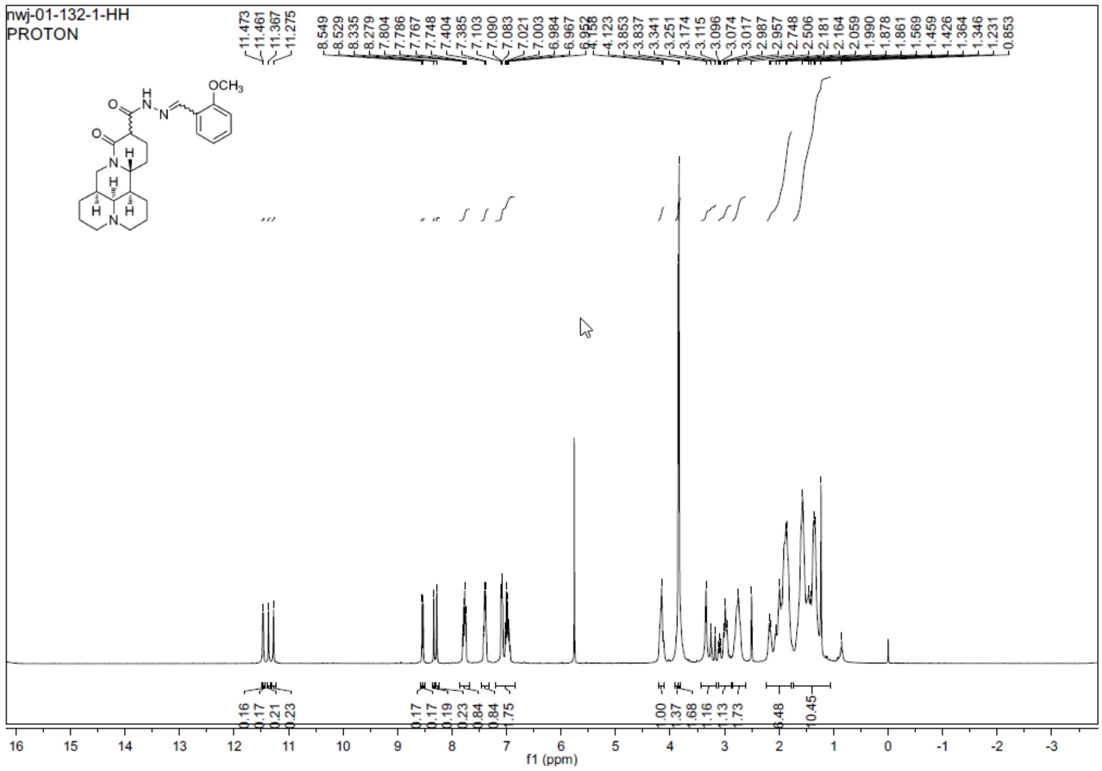

^{1}H NMR of 2

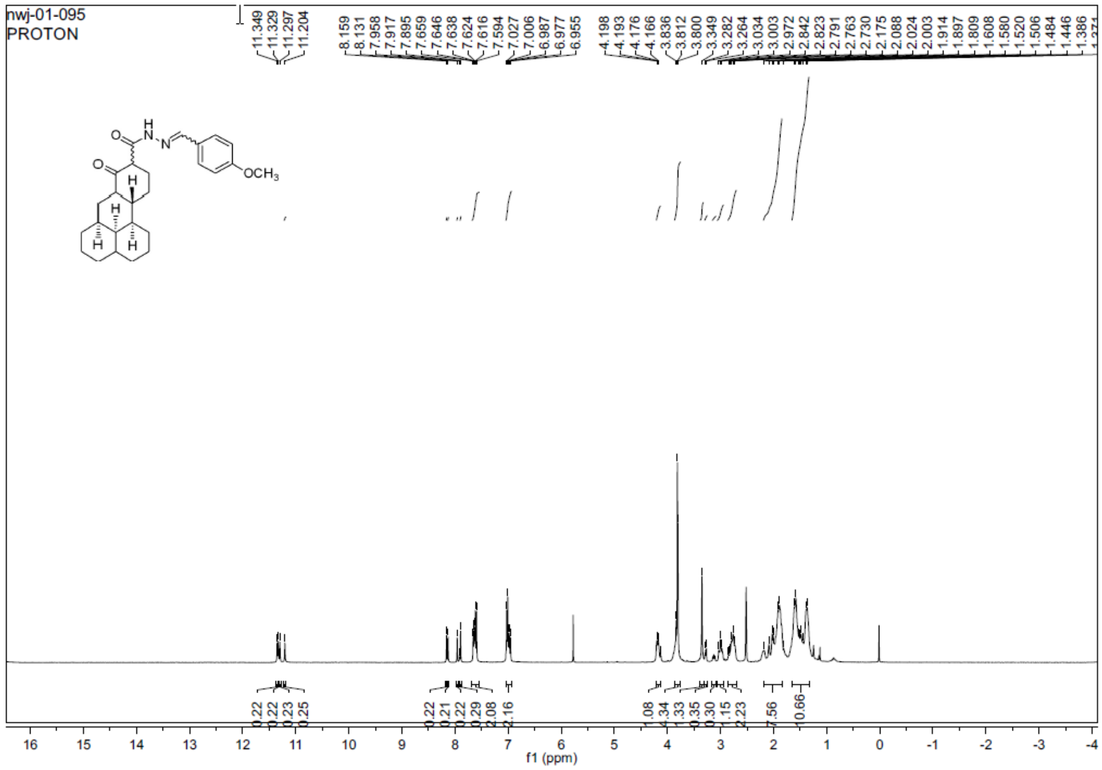

^{1}H NMR of 3

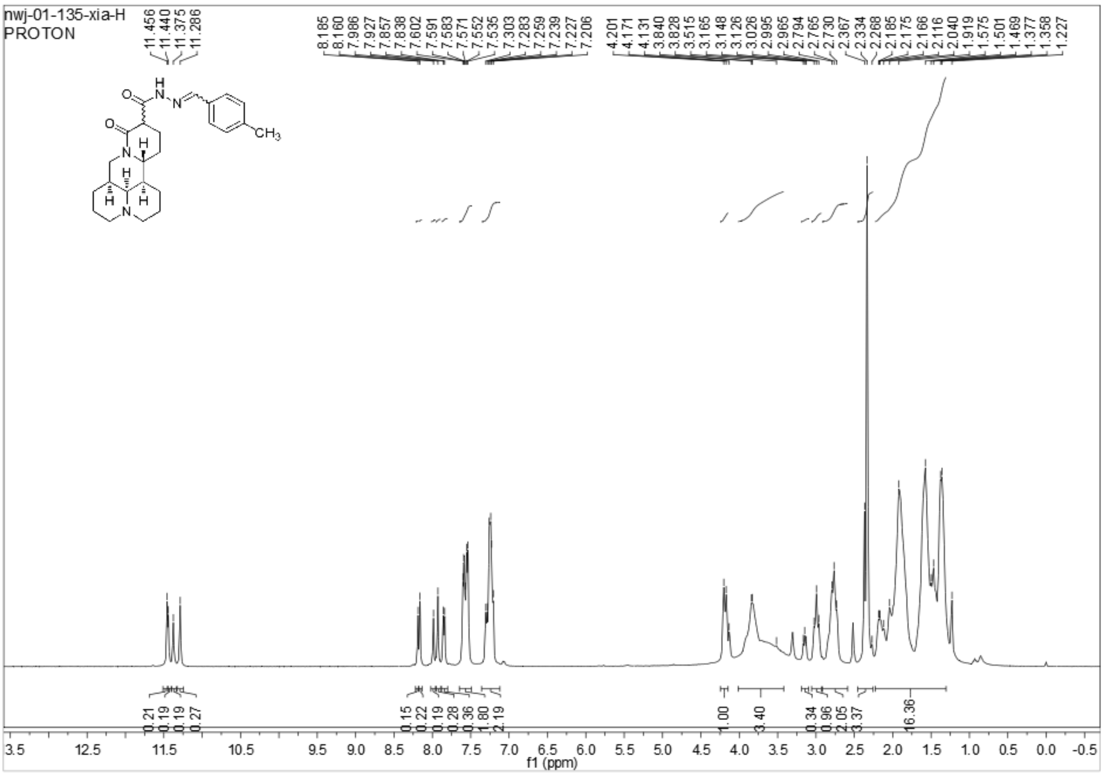

^{1}H NMR of 4

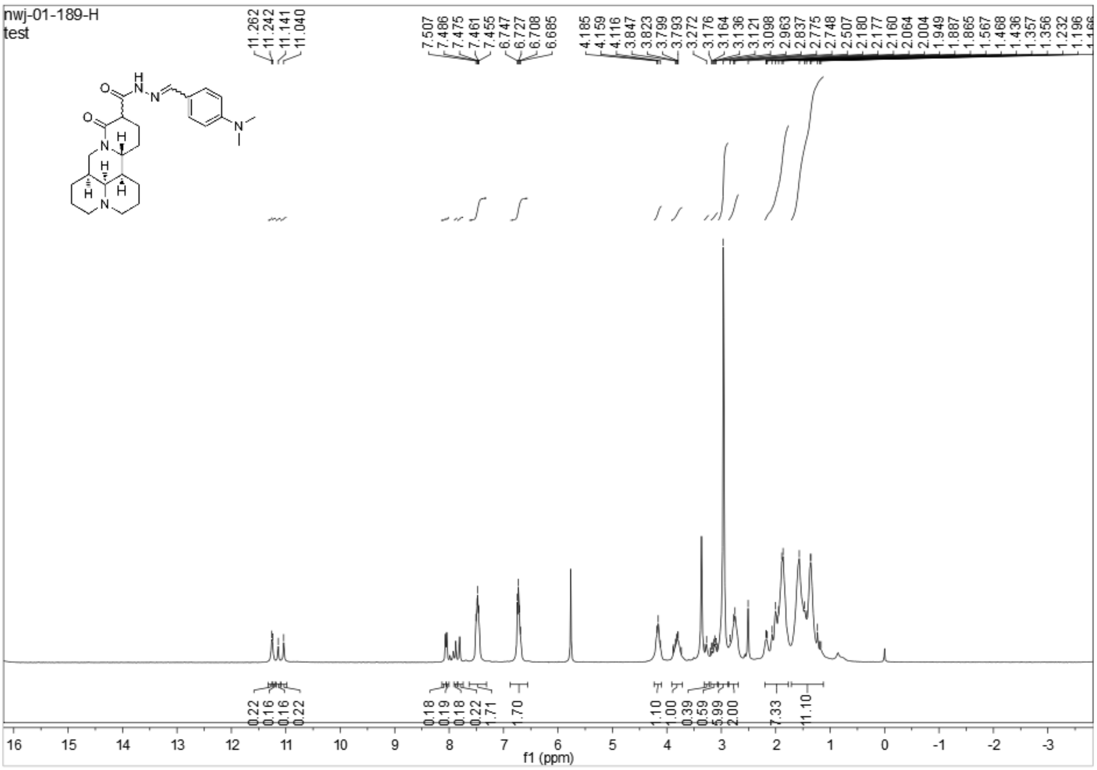

^{1}H NMR of 5

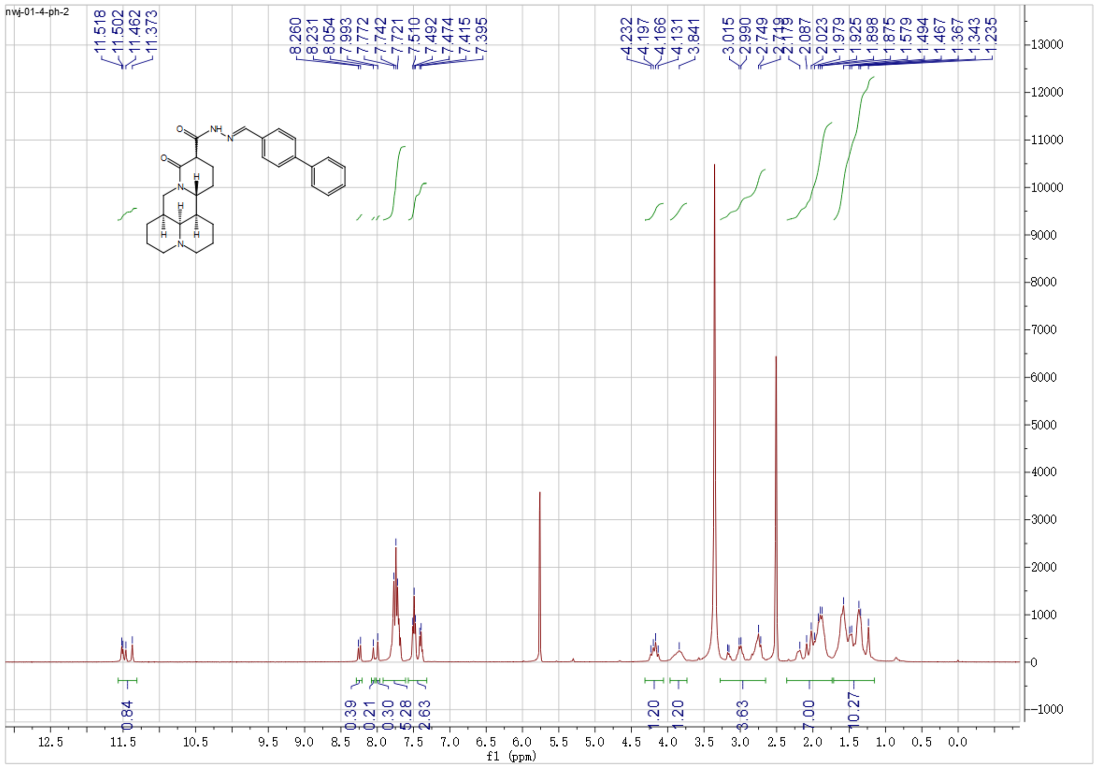

^{1}H NMR of 6

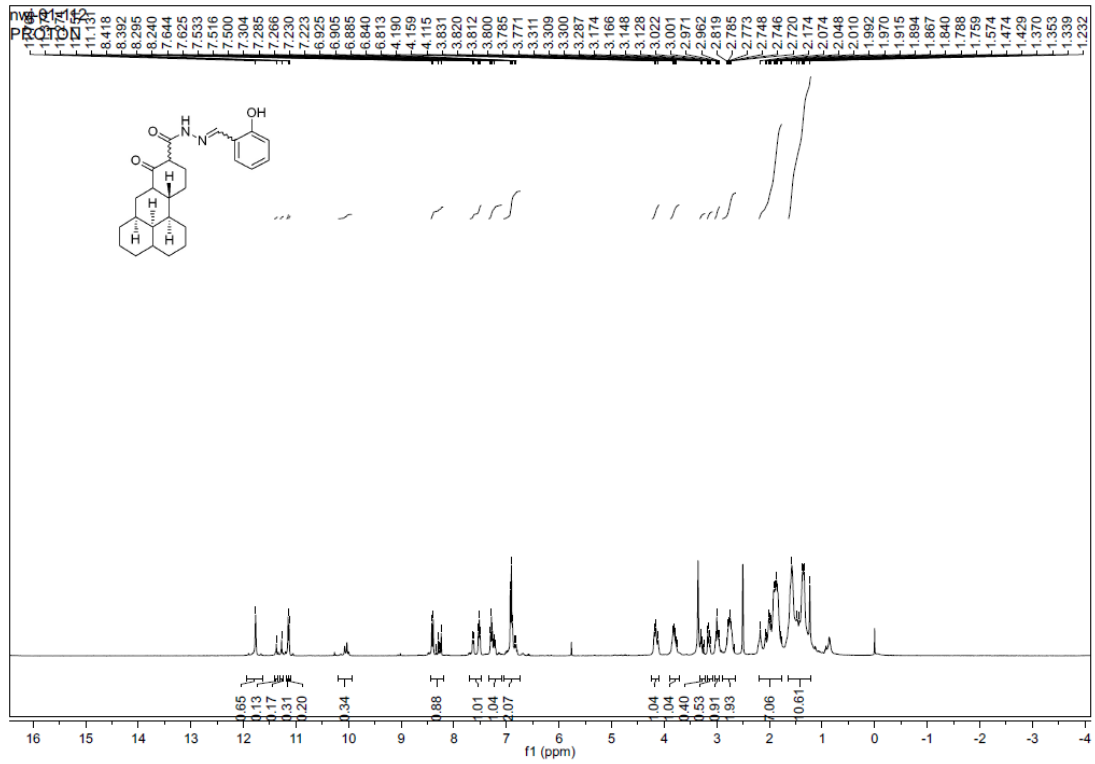

^{1}H NMR of 7

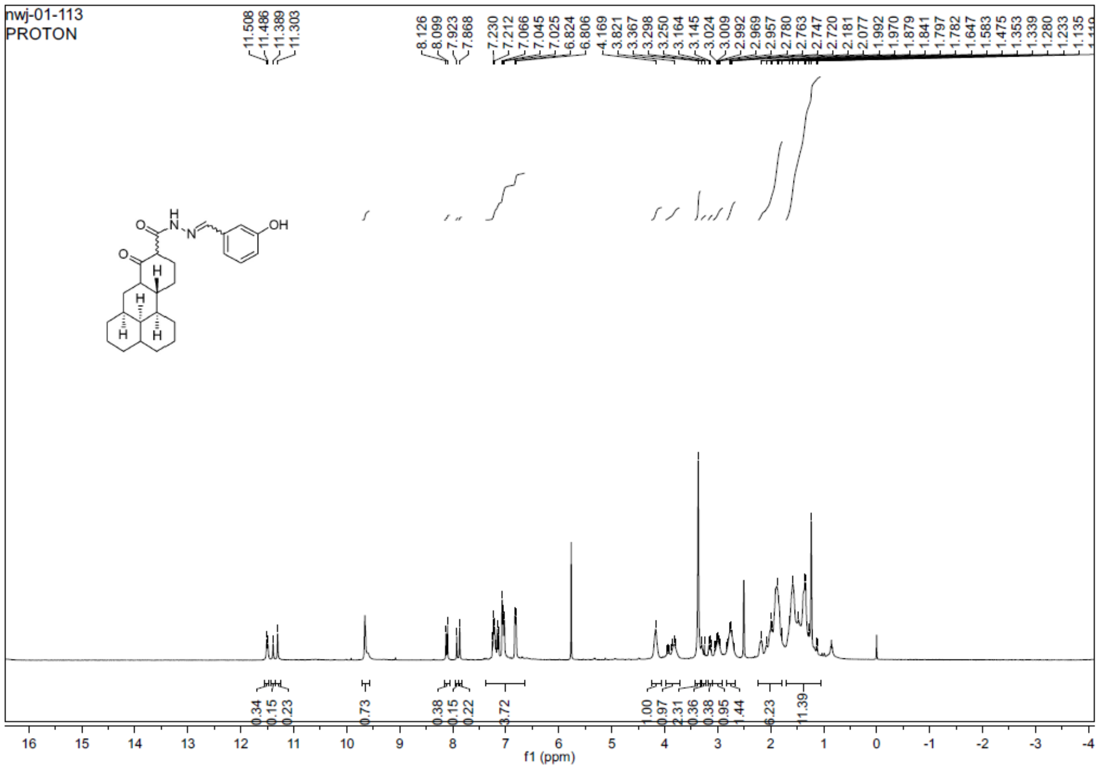

^{1}H NMR of 8

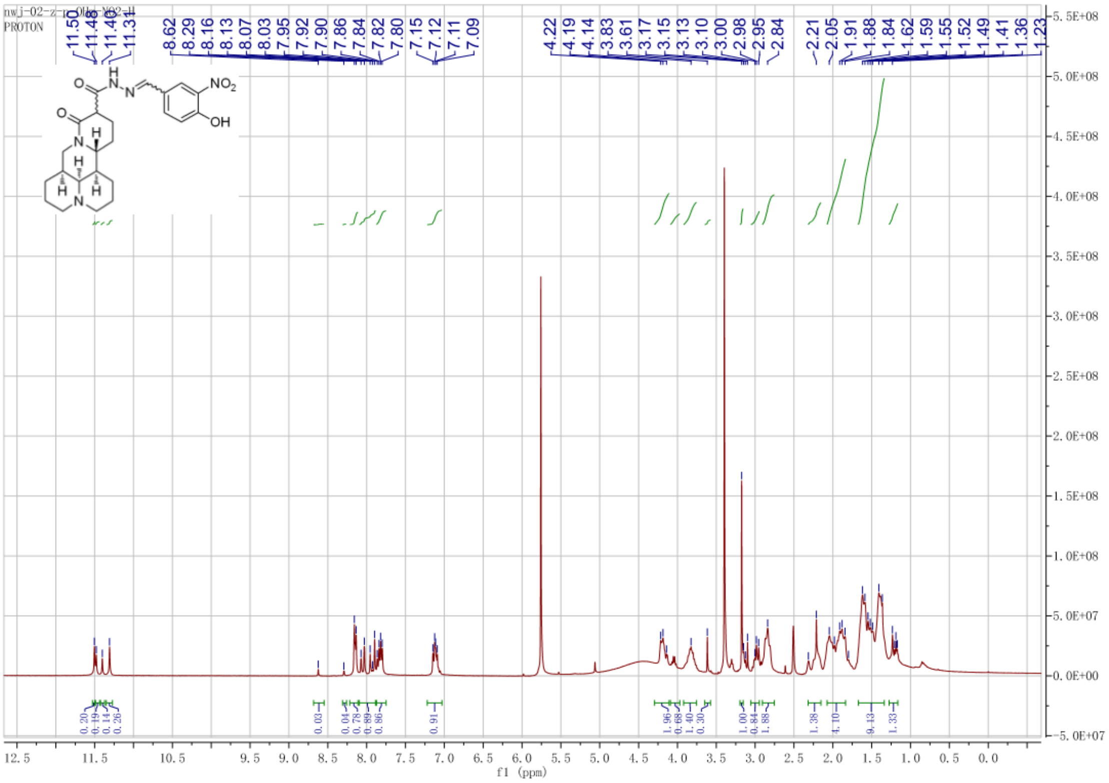

^{1}H NMR of 9

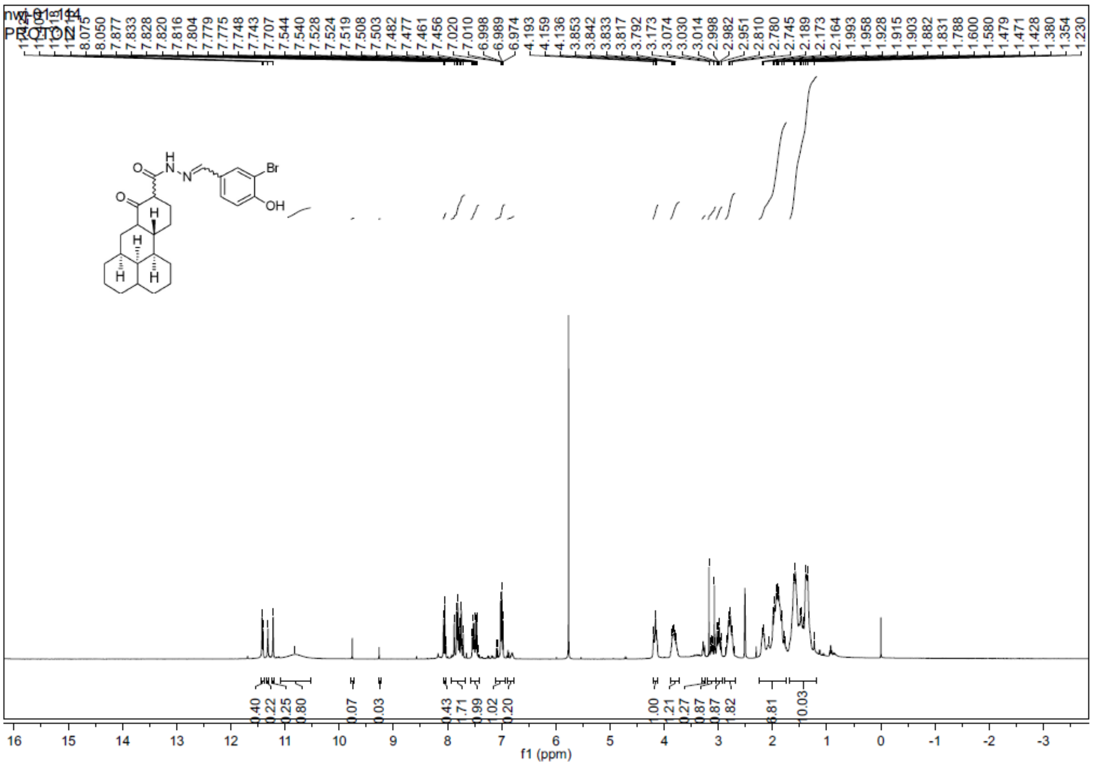

^{1}H NMR of 10

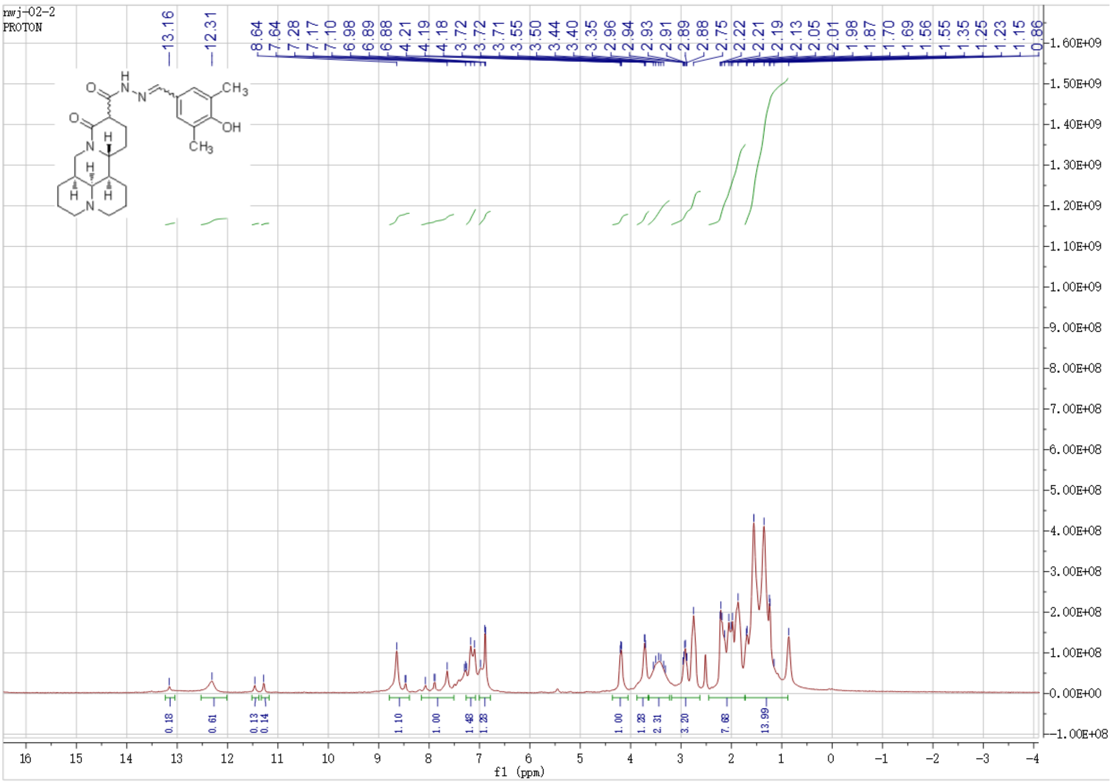

^{1}H NMR of 11

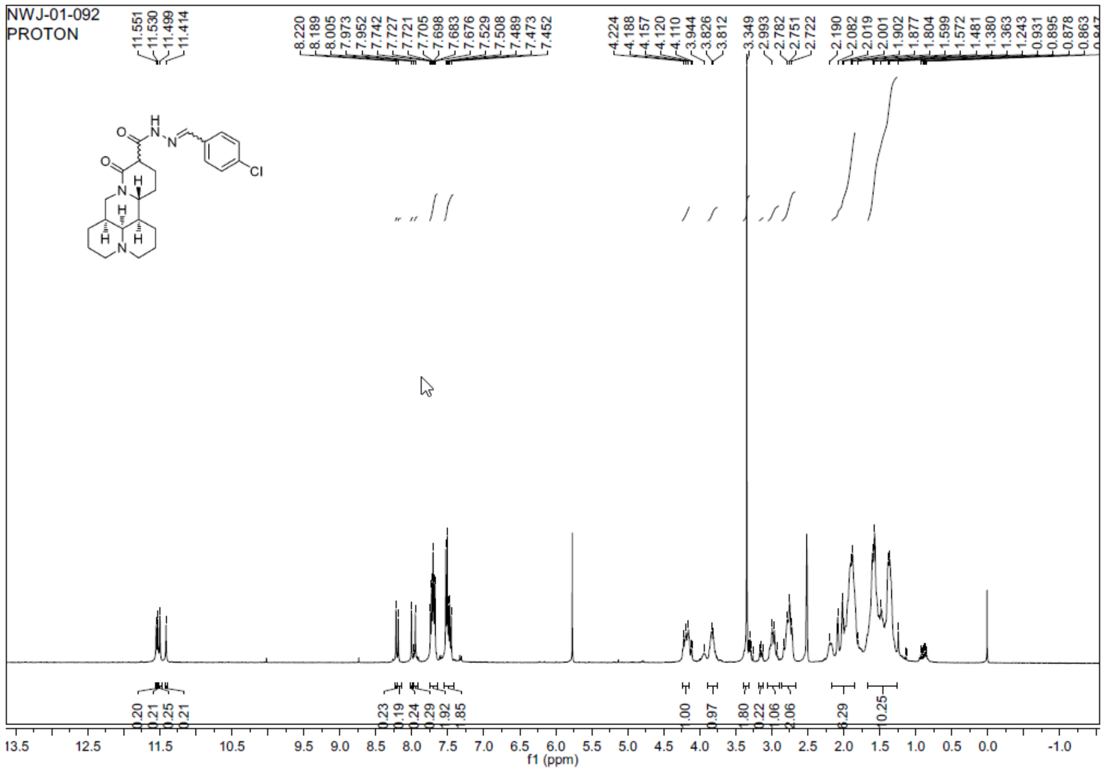

^{1}H NMR of 12

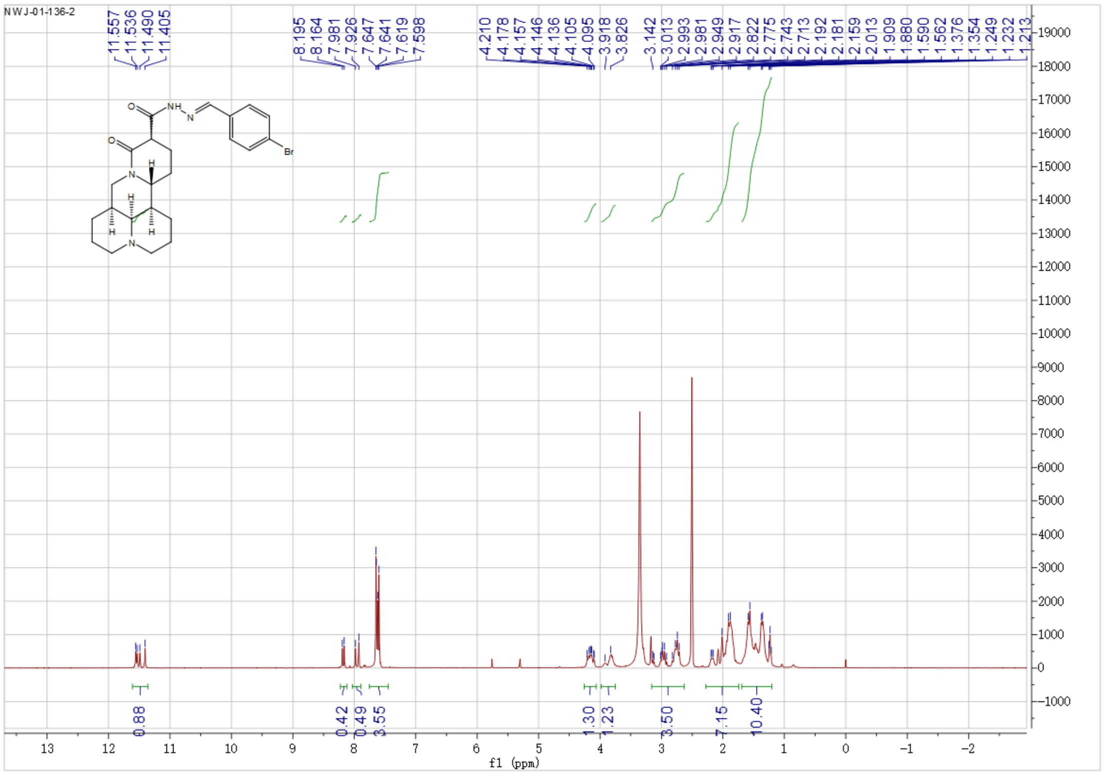

^{1}H NMR of 13

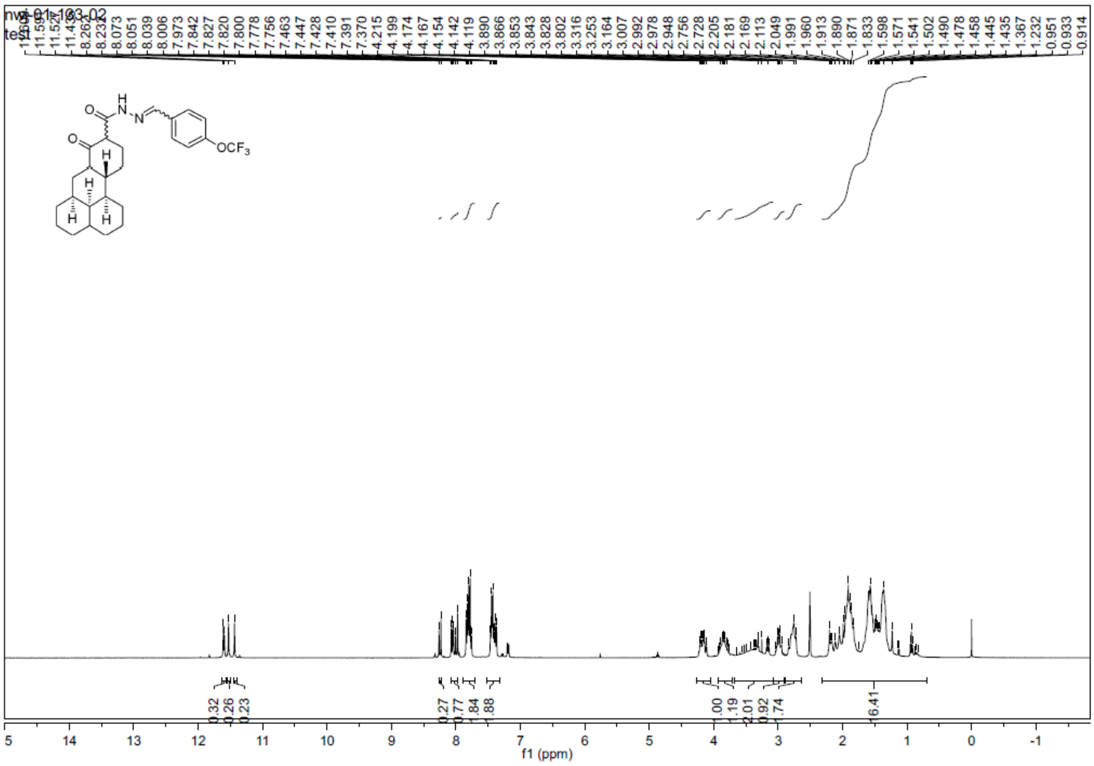

^{1}H NMR of 14

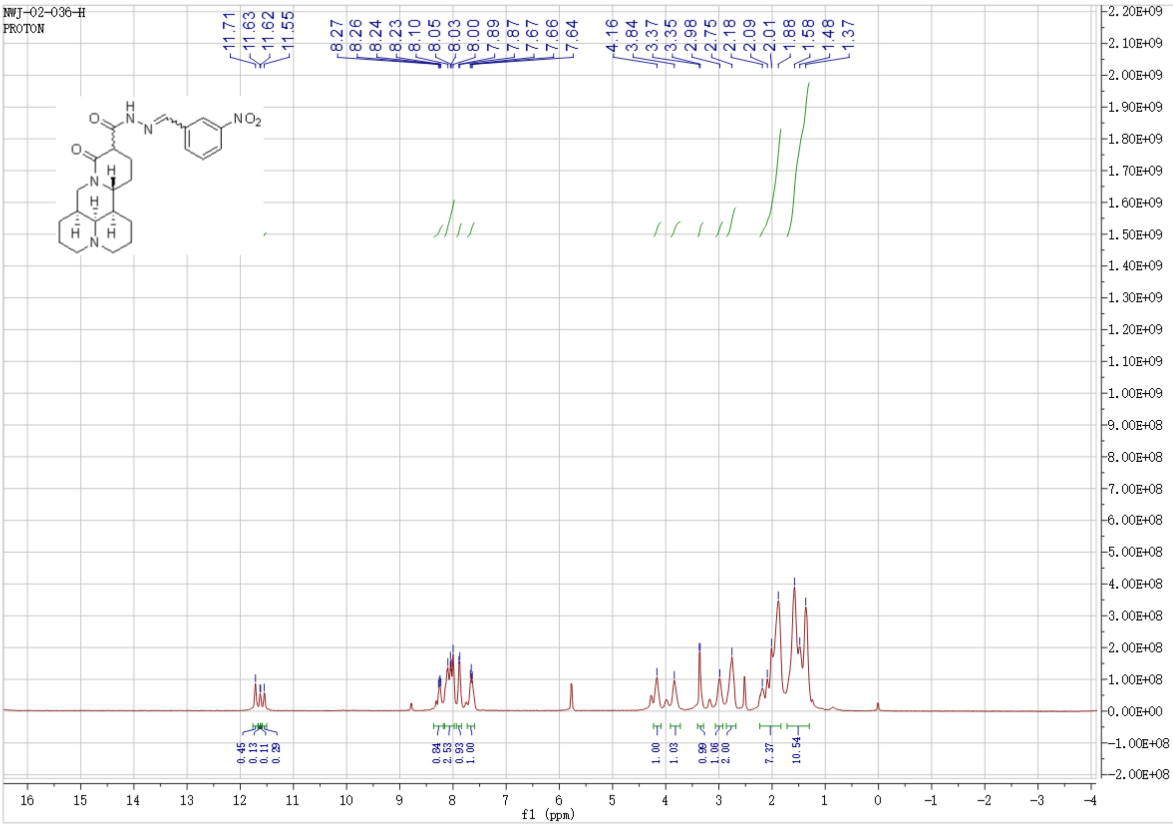

^{1}H NMR of 15

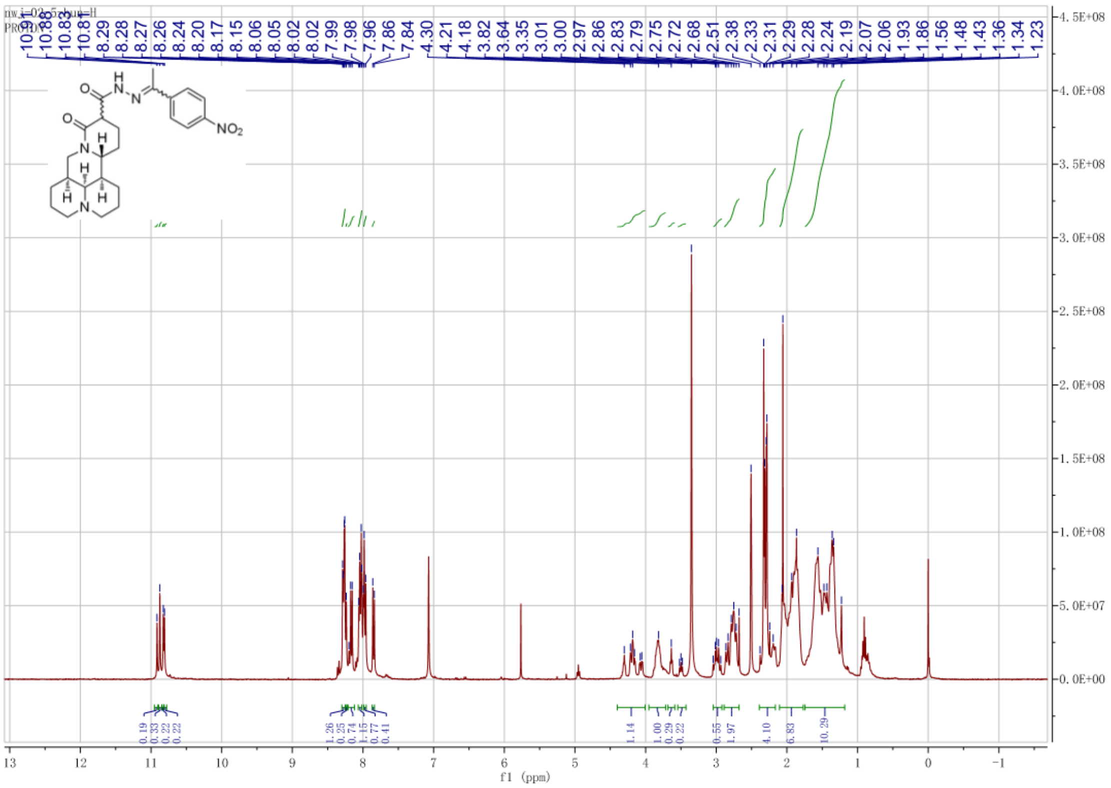

^{1}H NMR of 16

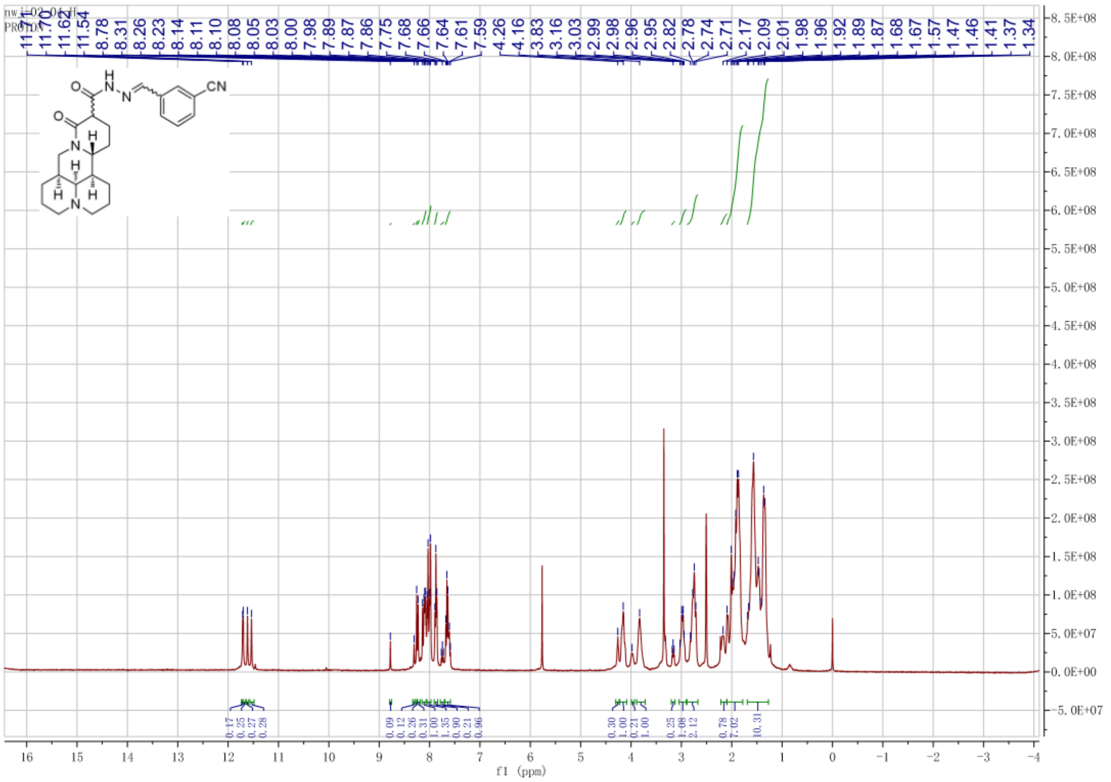

^{1}H NMR of 17

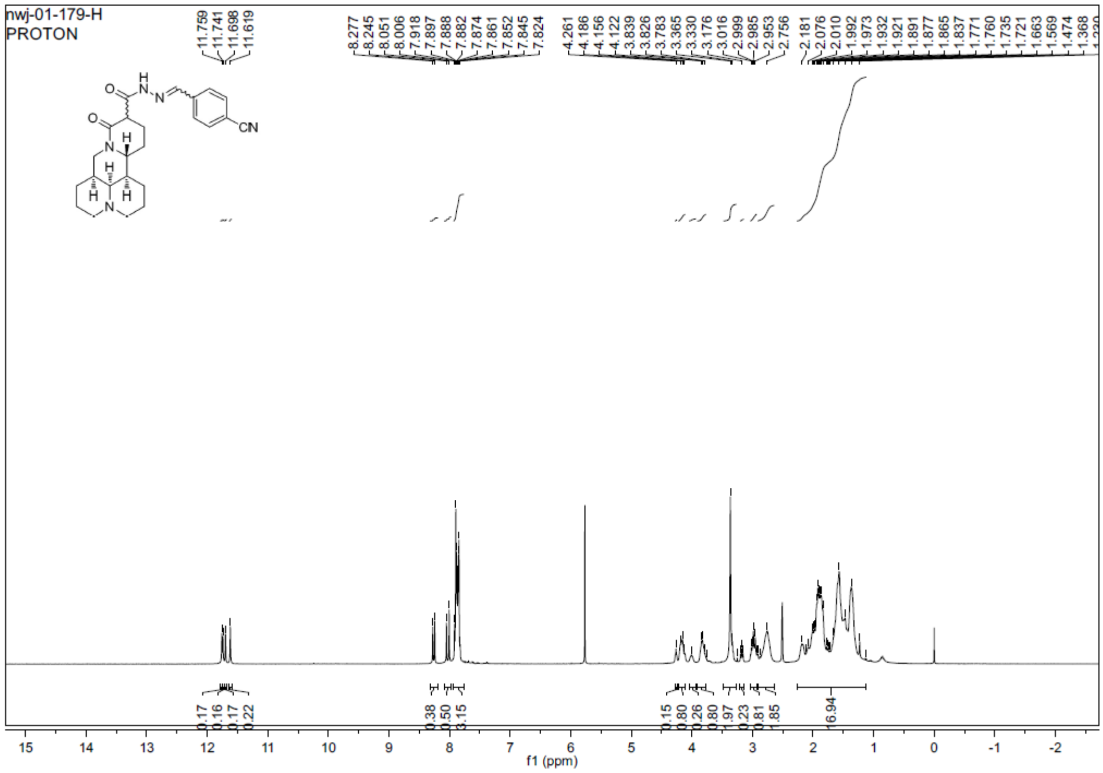

^{1}H NMR of 18

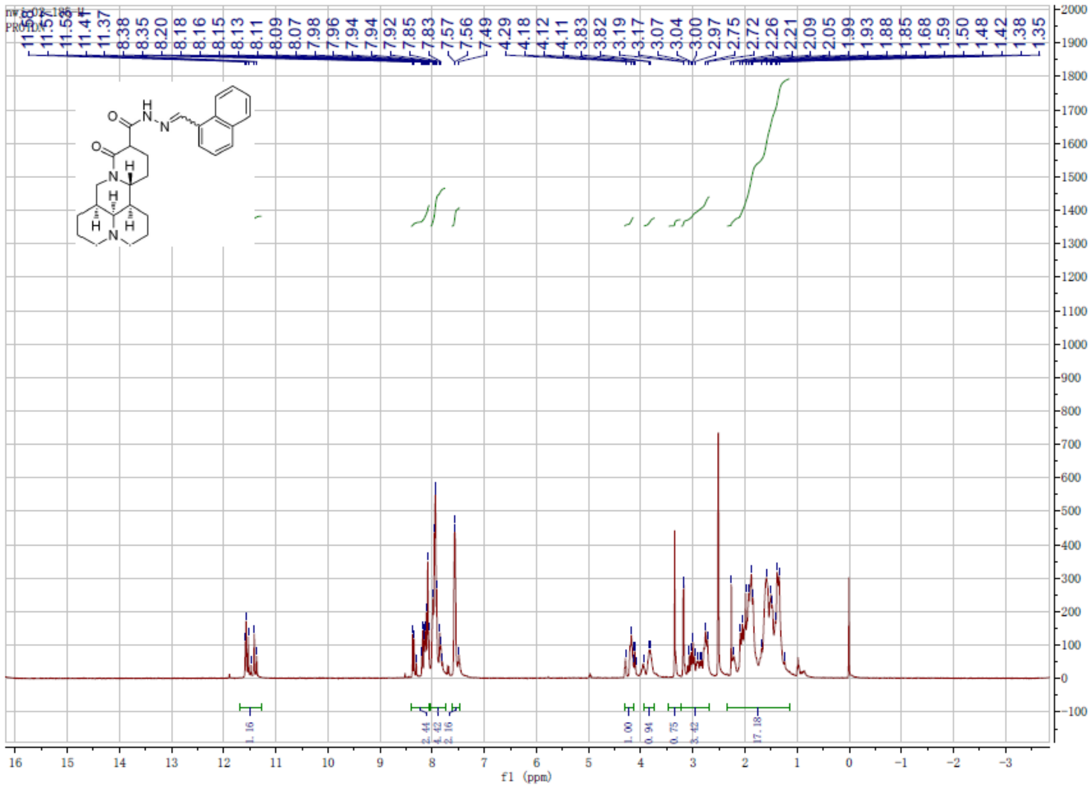

^{1}H NMR of 19

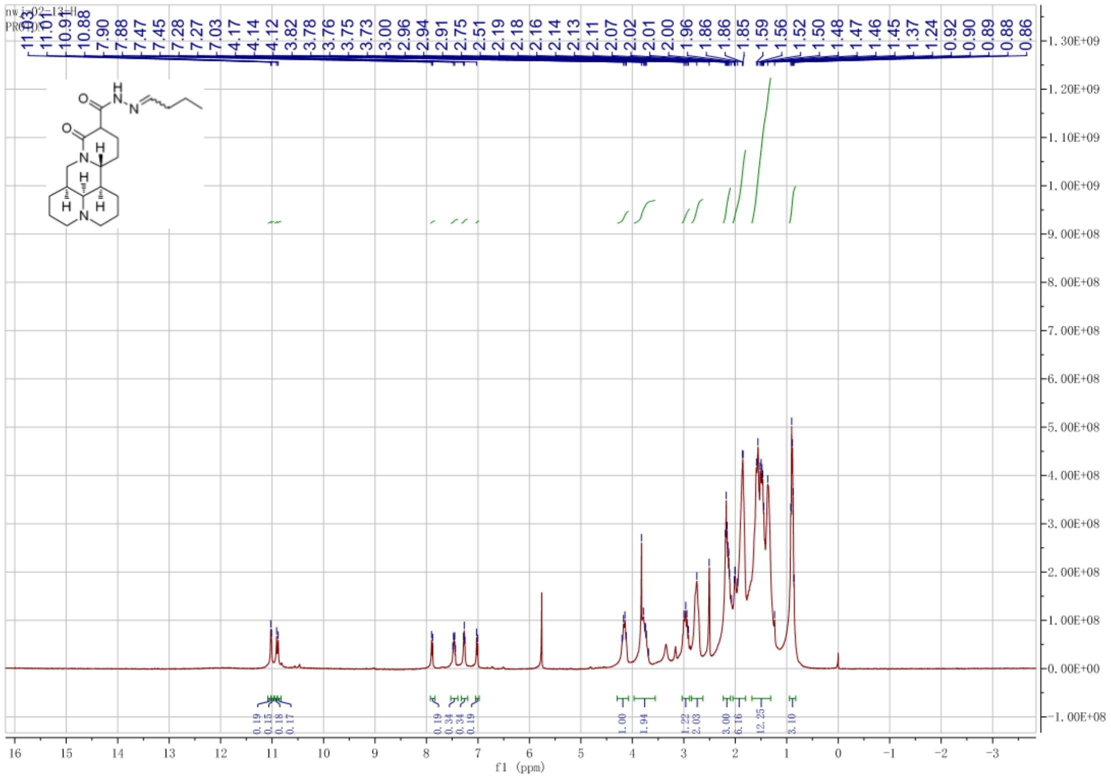

^{1}H NMR of 20

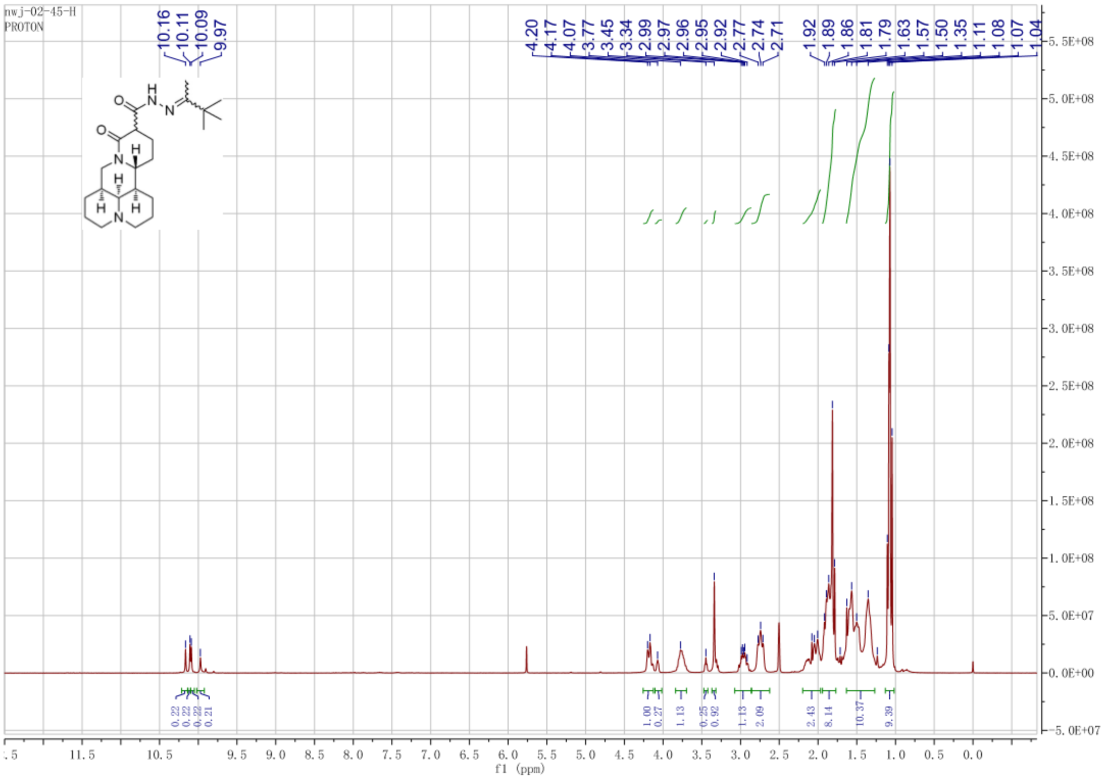

^{1}H NMR of 21

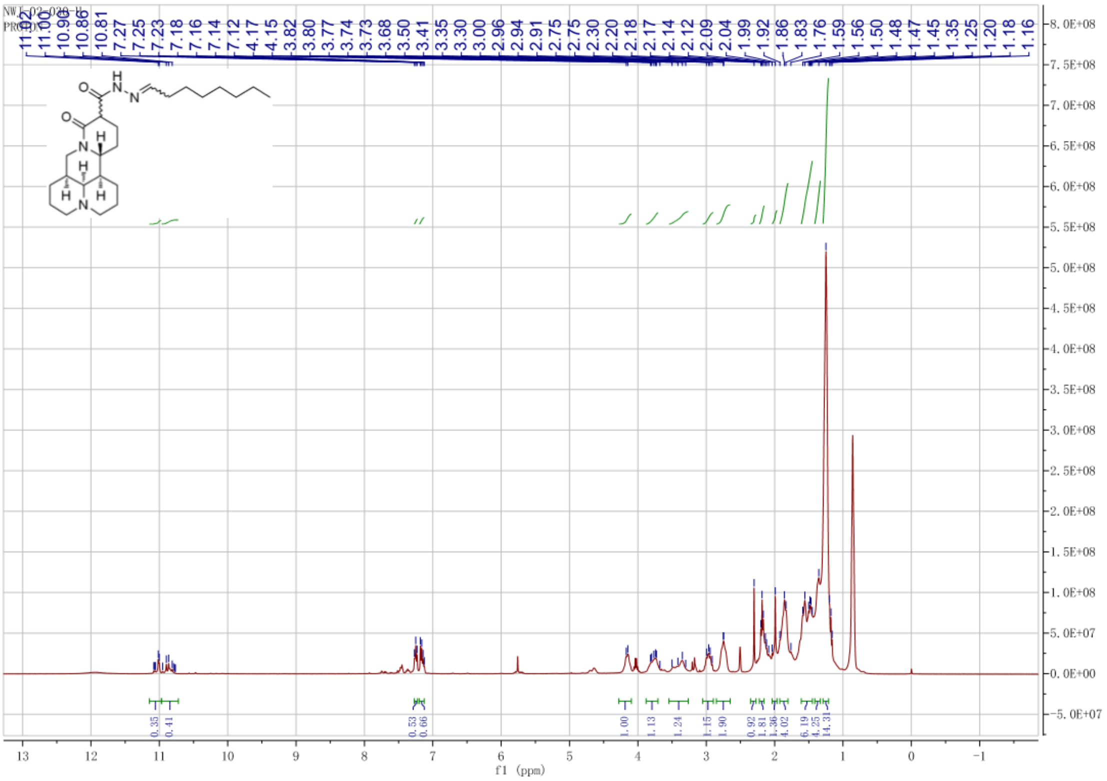

^{1}H NMR of 22

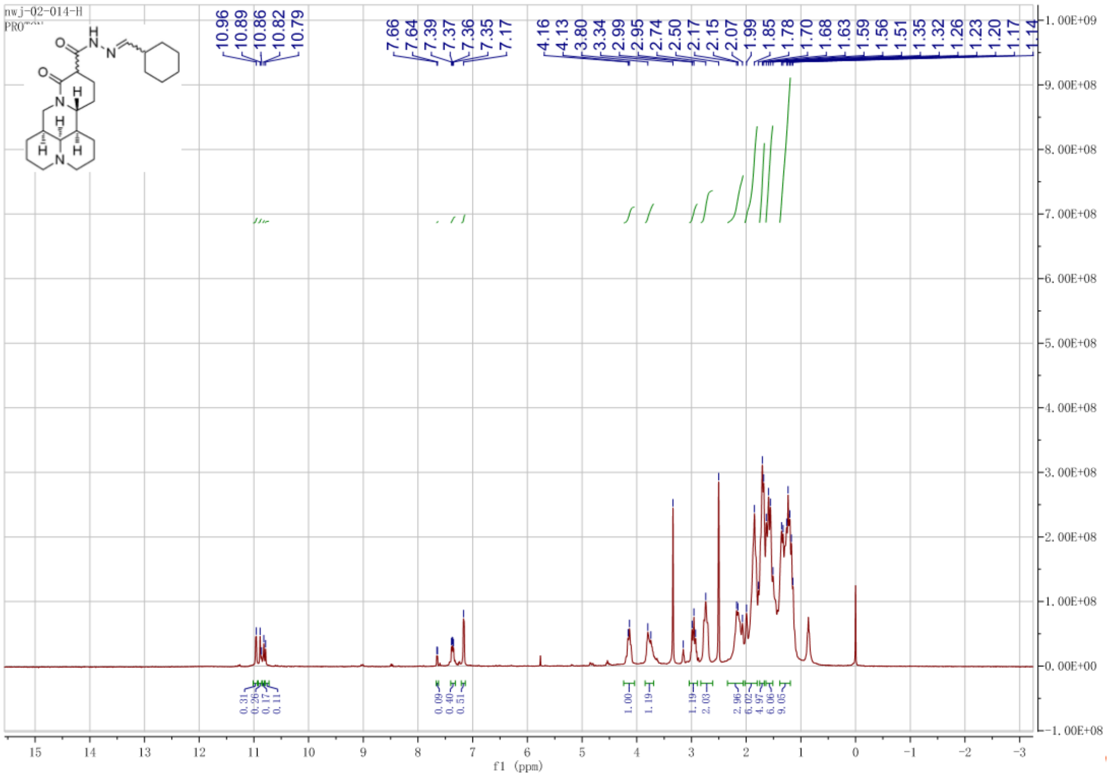

^{1}H NMR of 23

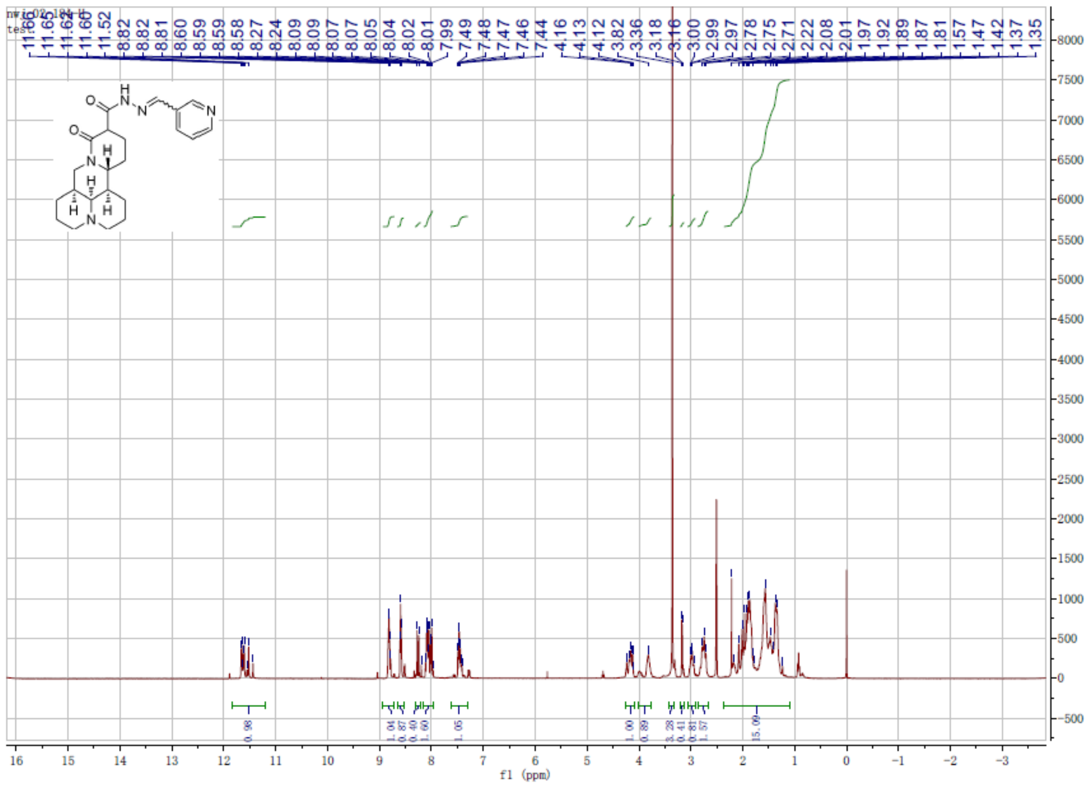

^{1}H NMR of 24

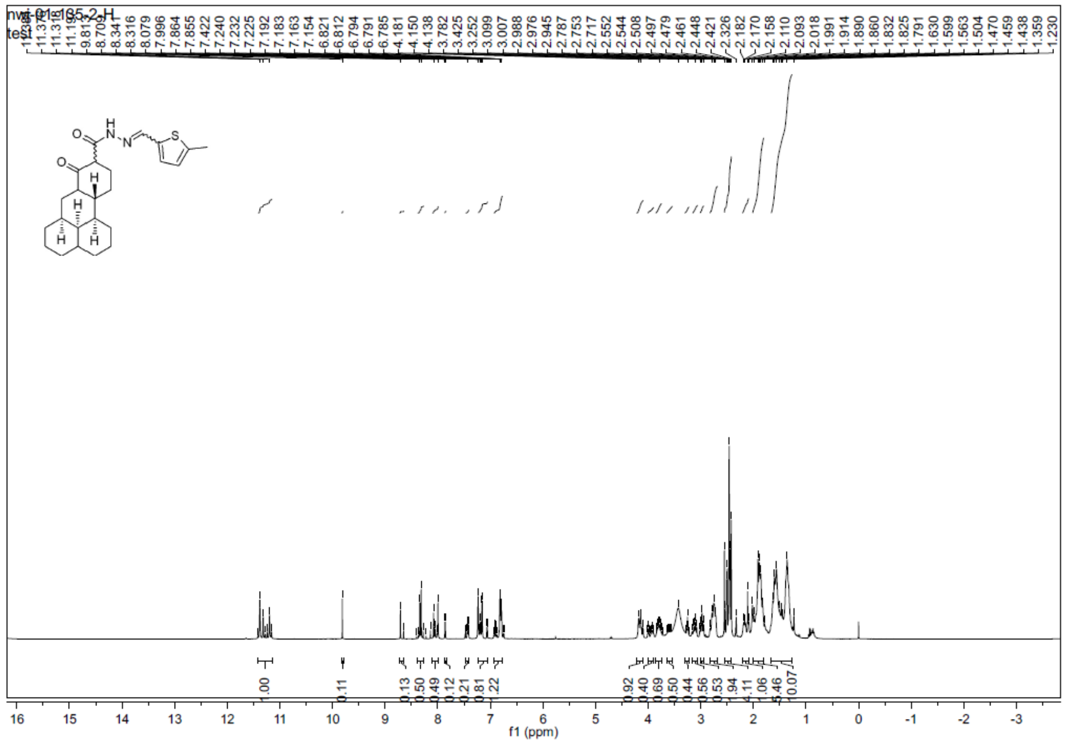

^{1}H NMR of 25

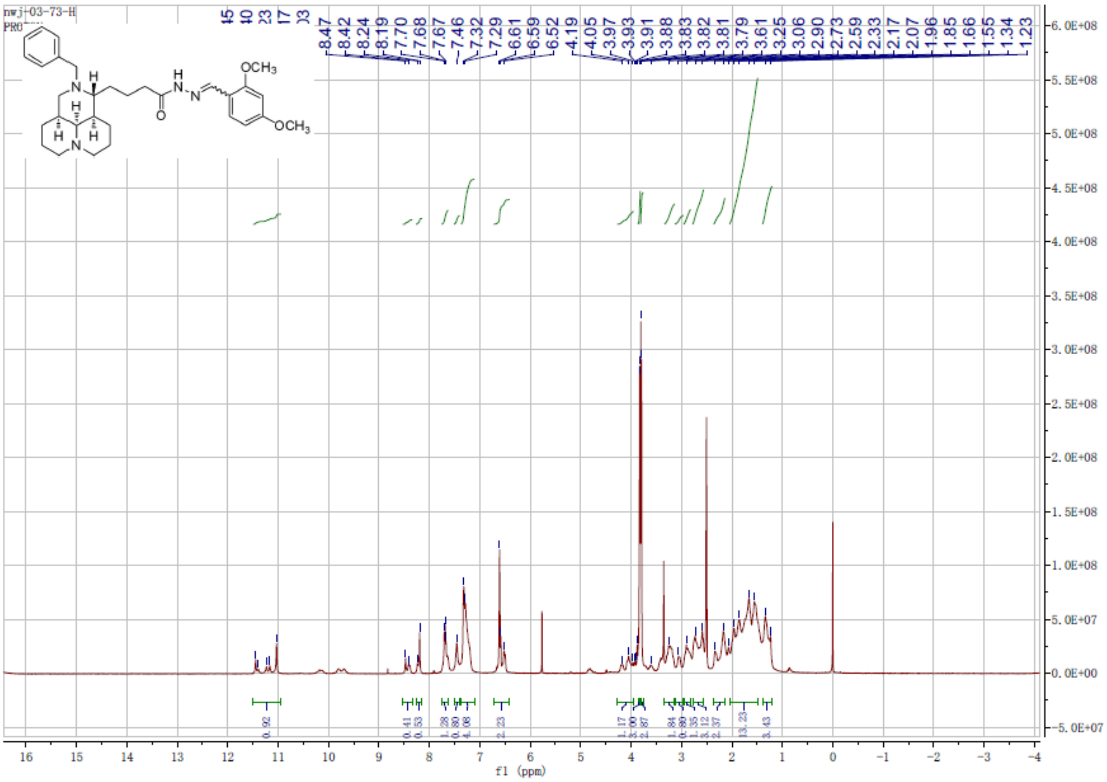

^{1}H NMR of 26

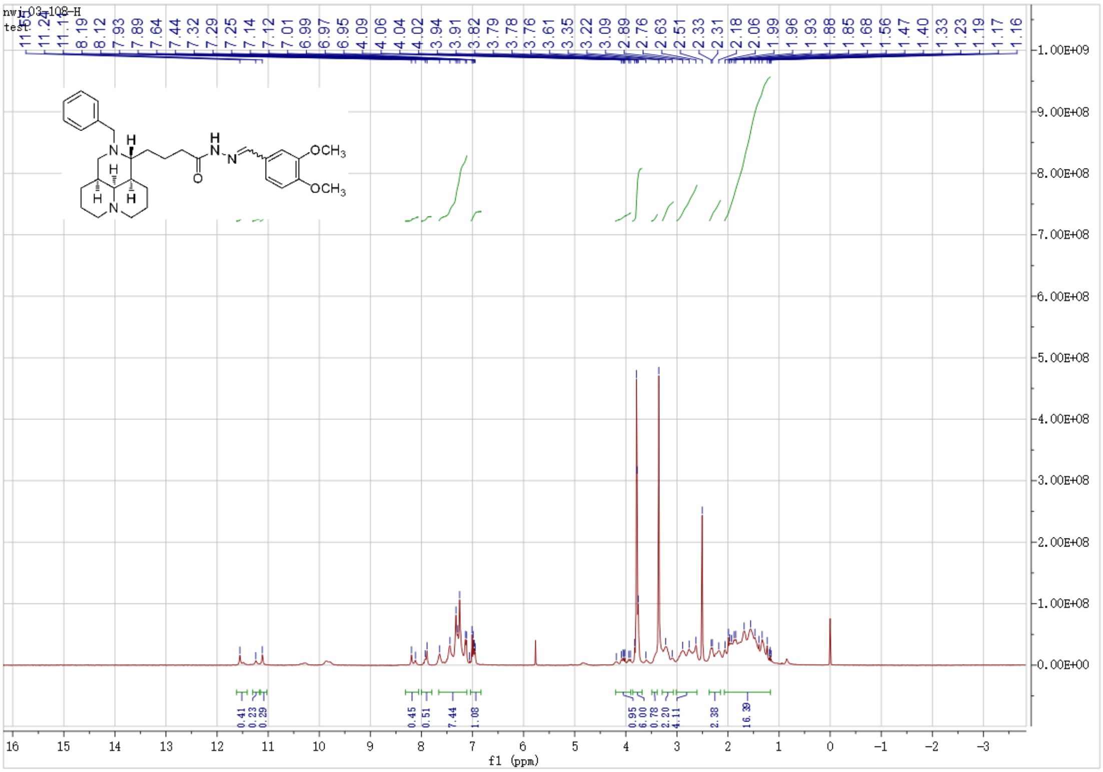

^{1}H NMR of 27

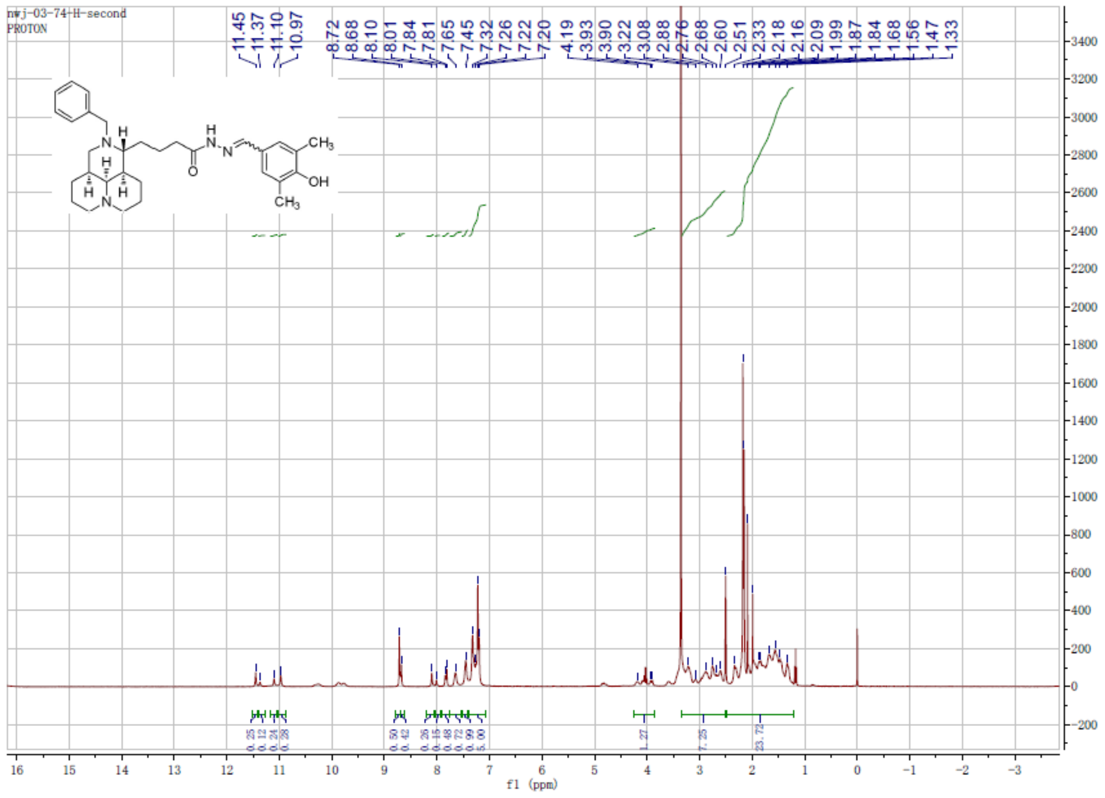

^{1}H NMR of 28

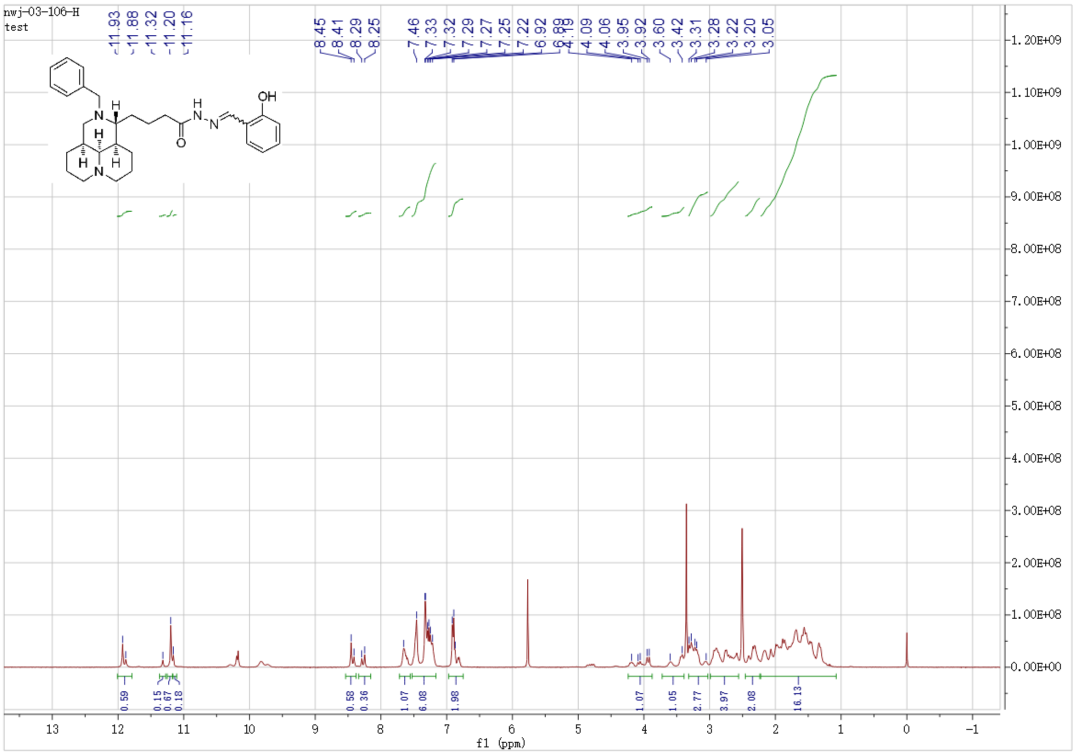

^{1}H NMR of 29

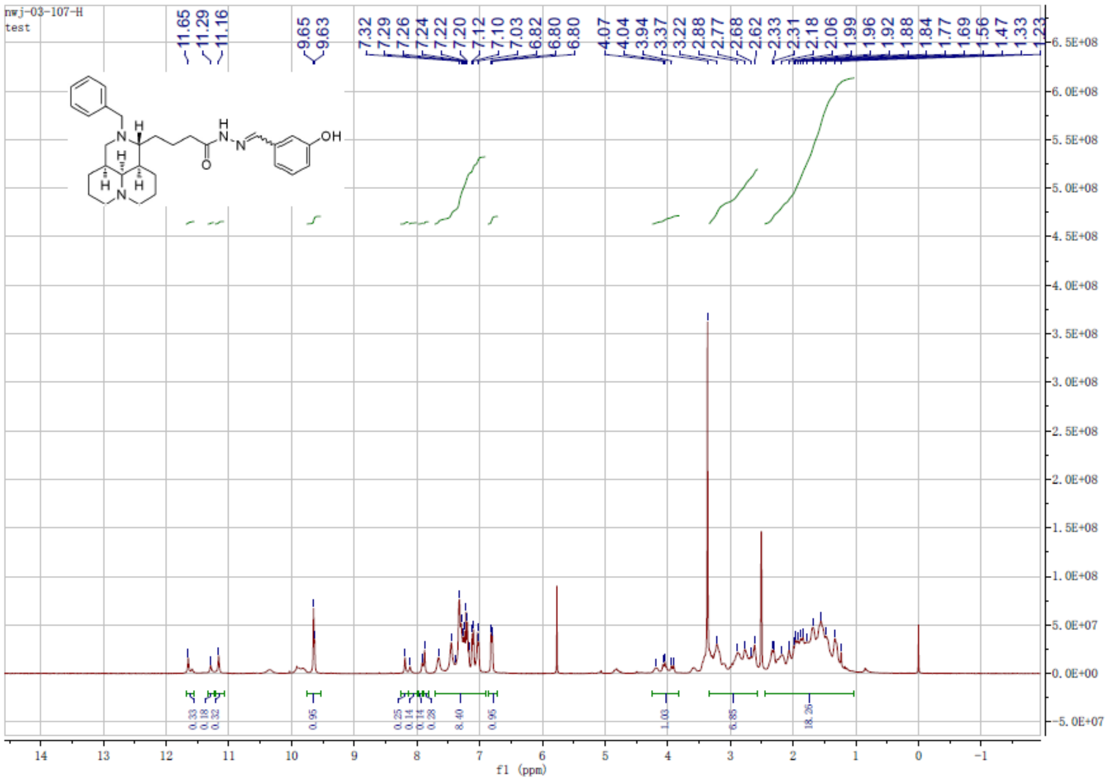

^{1}H NMR of 30

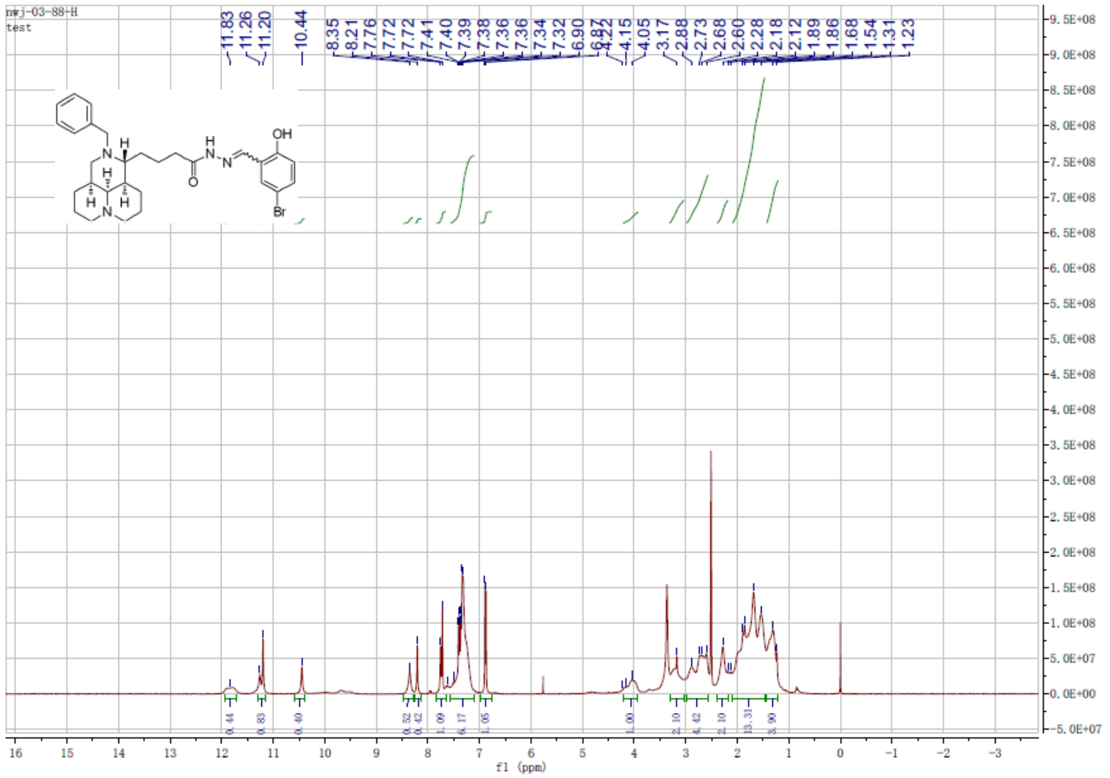

^{1}H NMR of 31

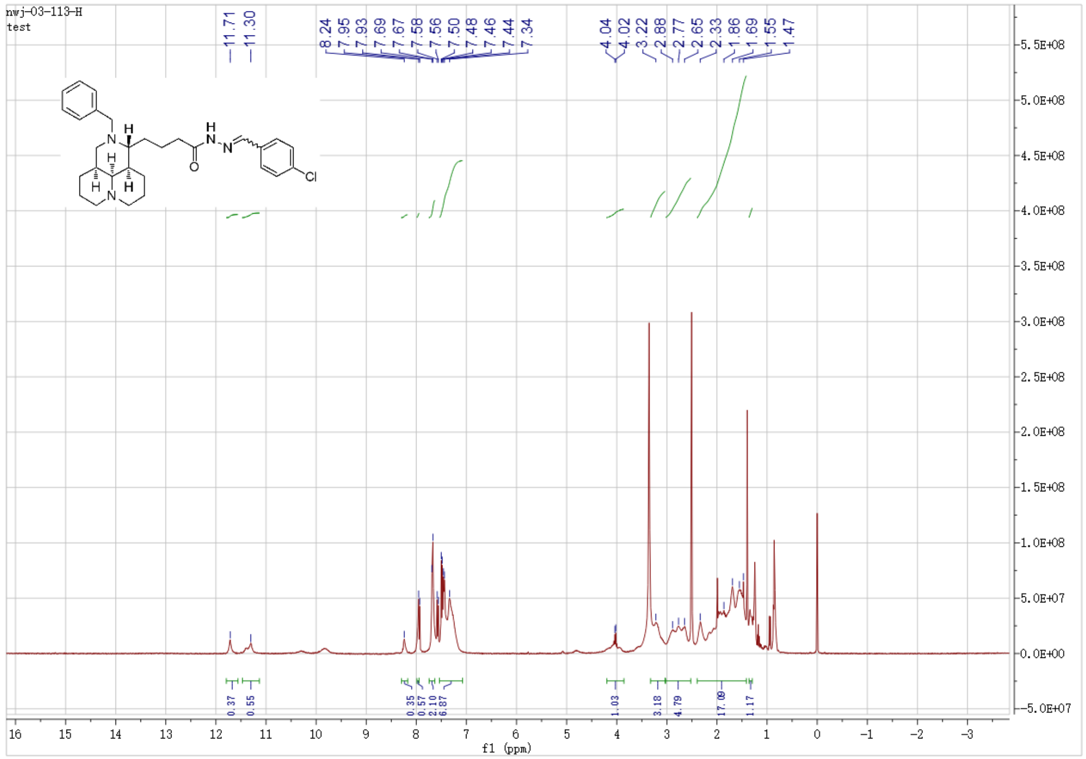

^{1}H NMR of 32

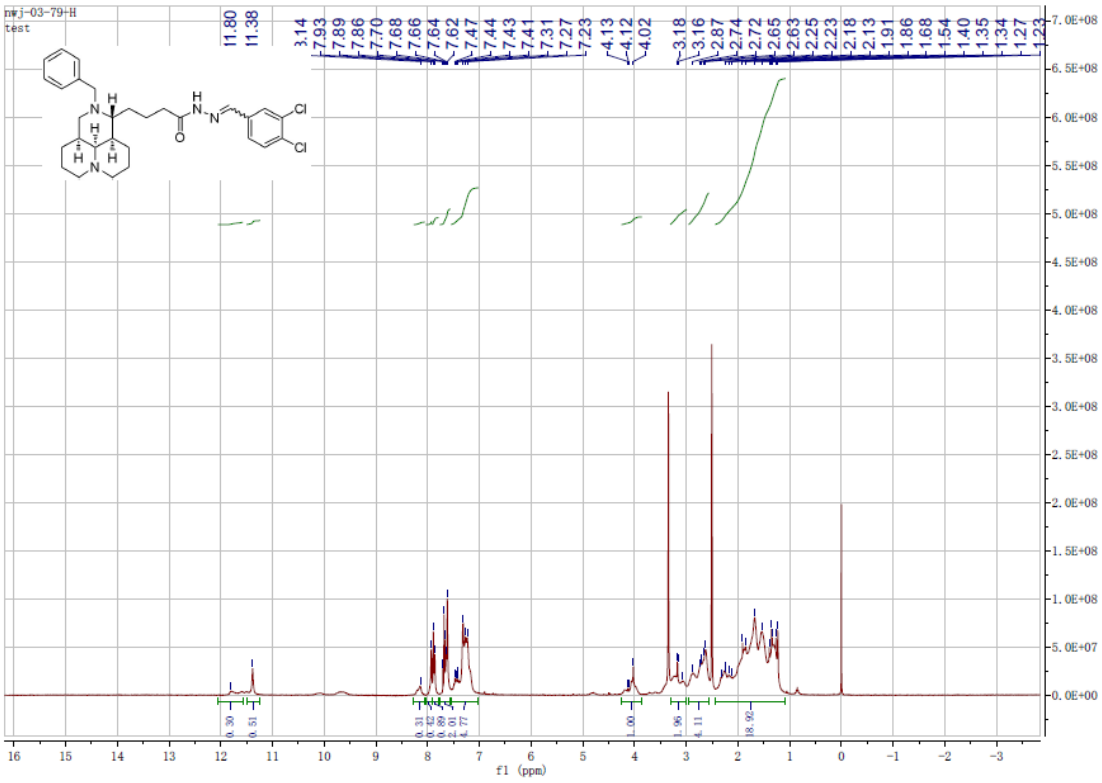

^{1}H NMR of 33

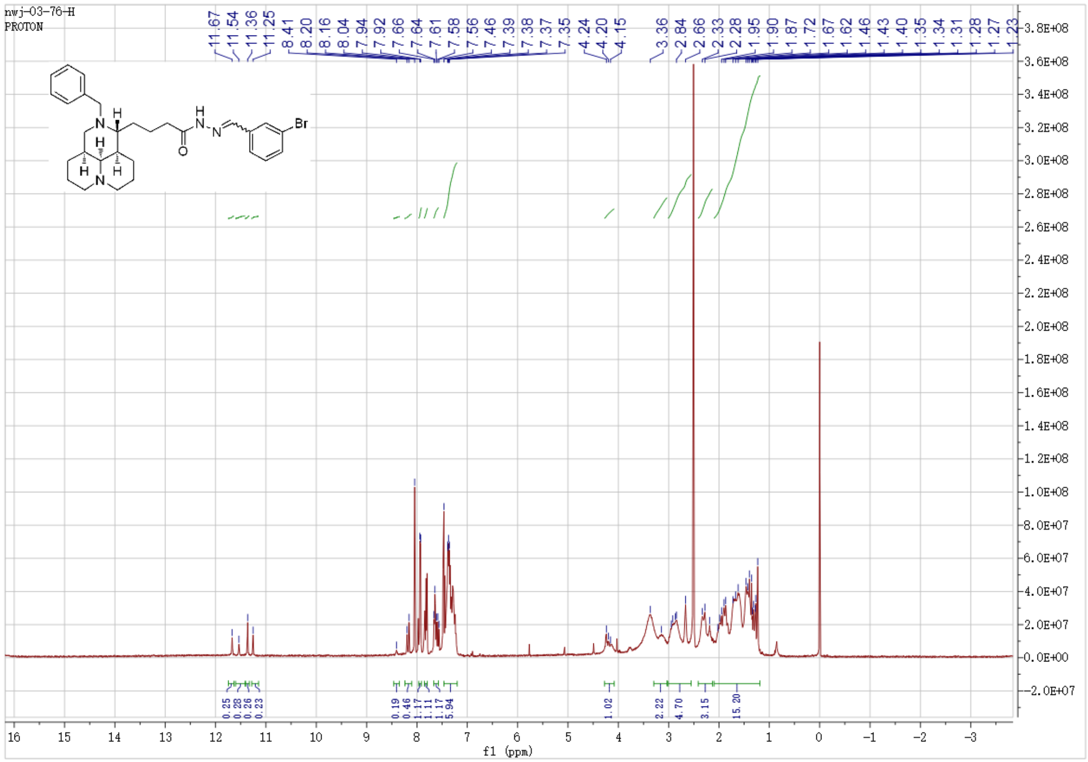

^{1}H NMR of 34

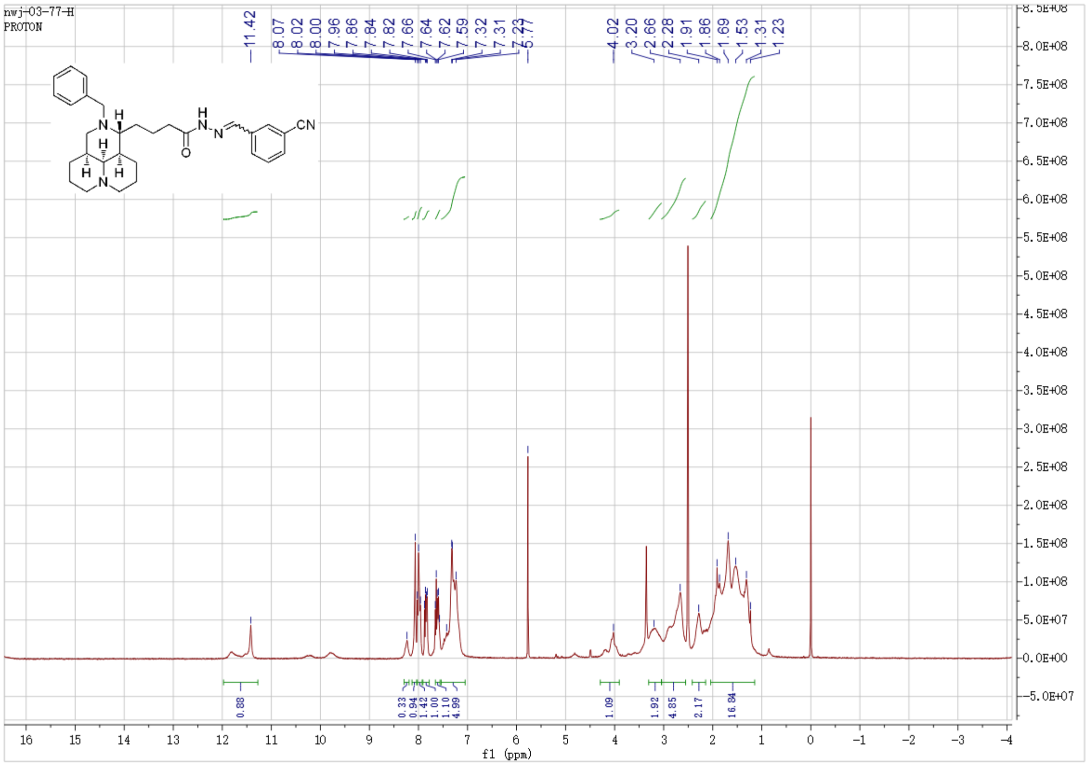

^{1}H NMR of 35

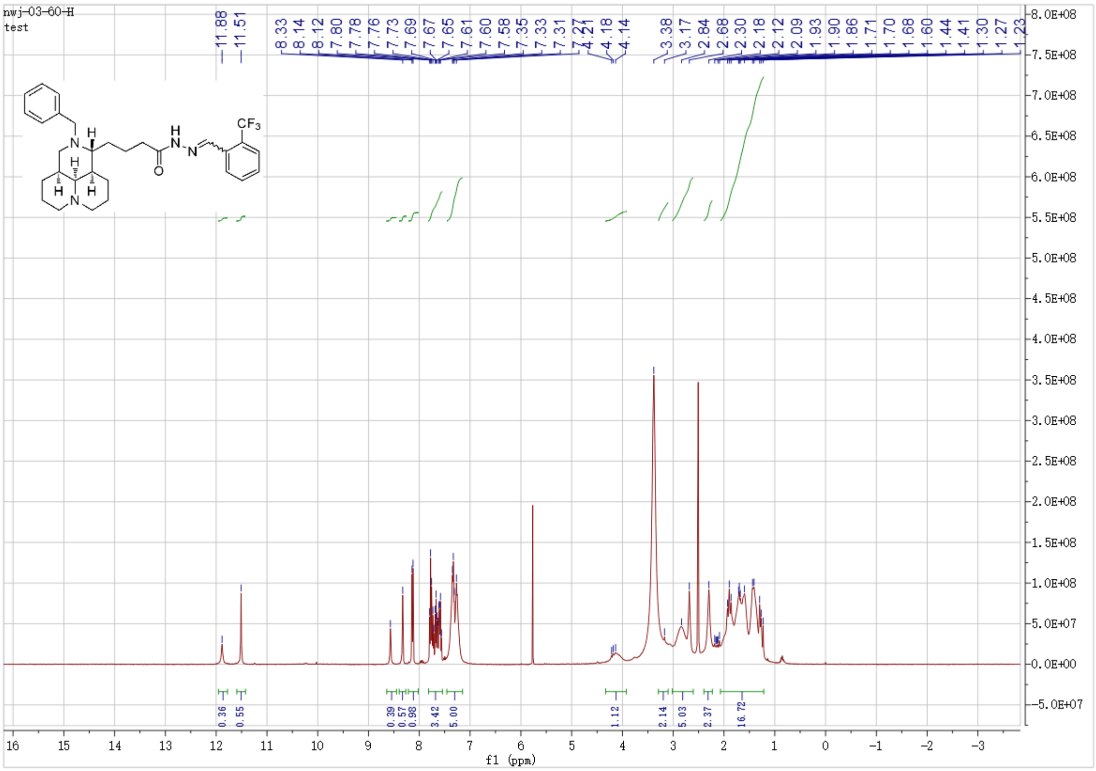

^{1}H NMR of 36

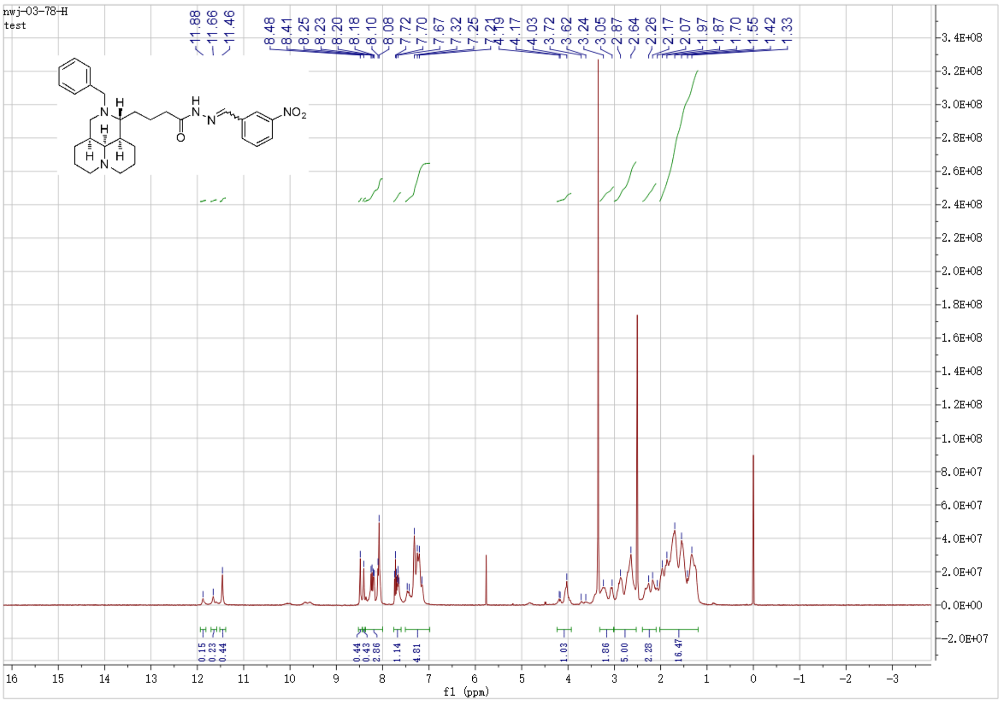

^{1}H NMR of 37

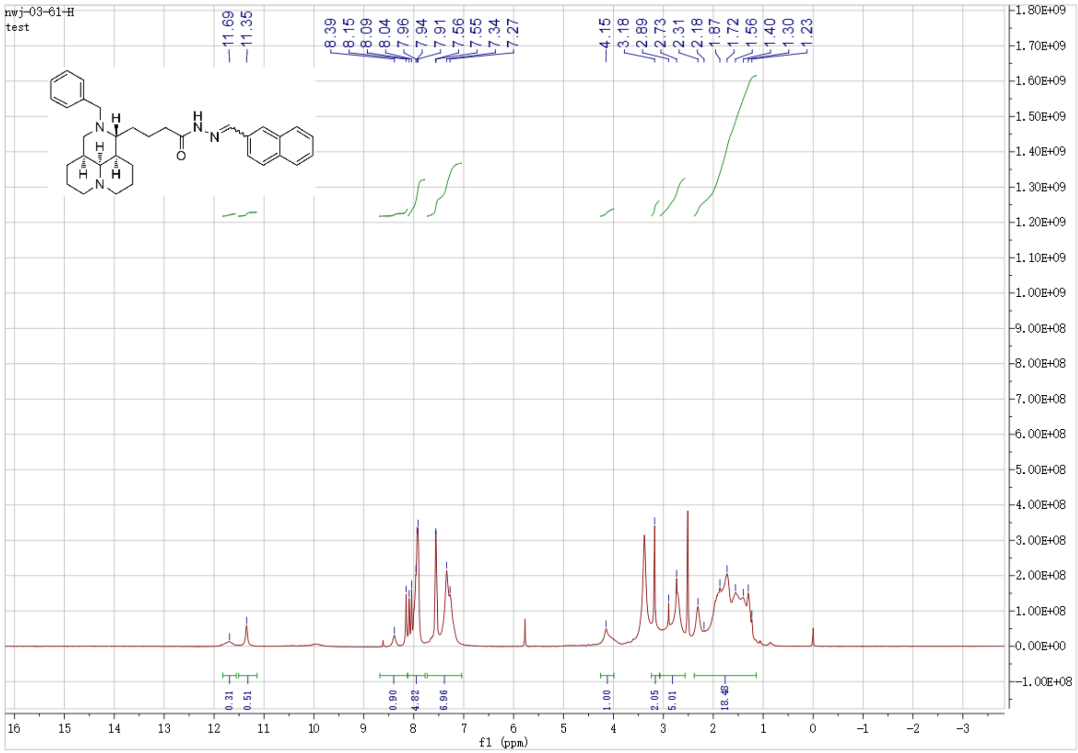

^{1}H NMR of 38

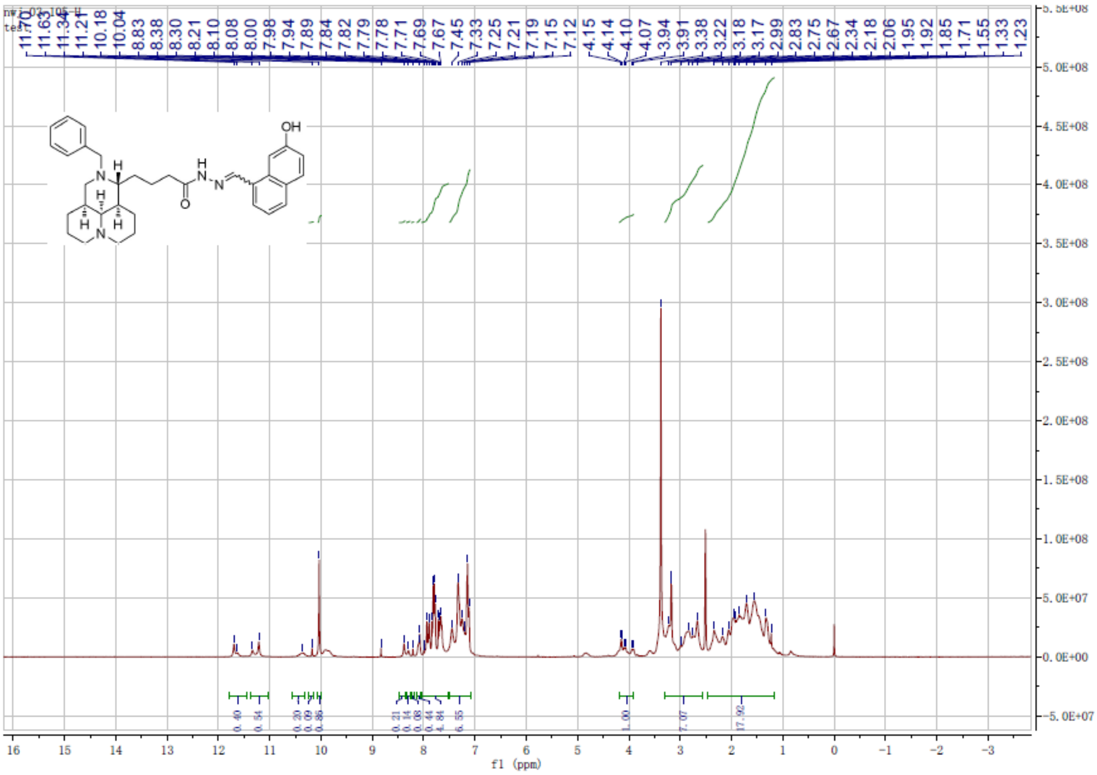

^{1}H NMR of 39

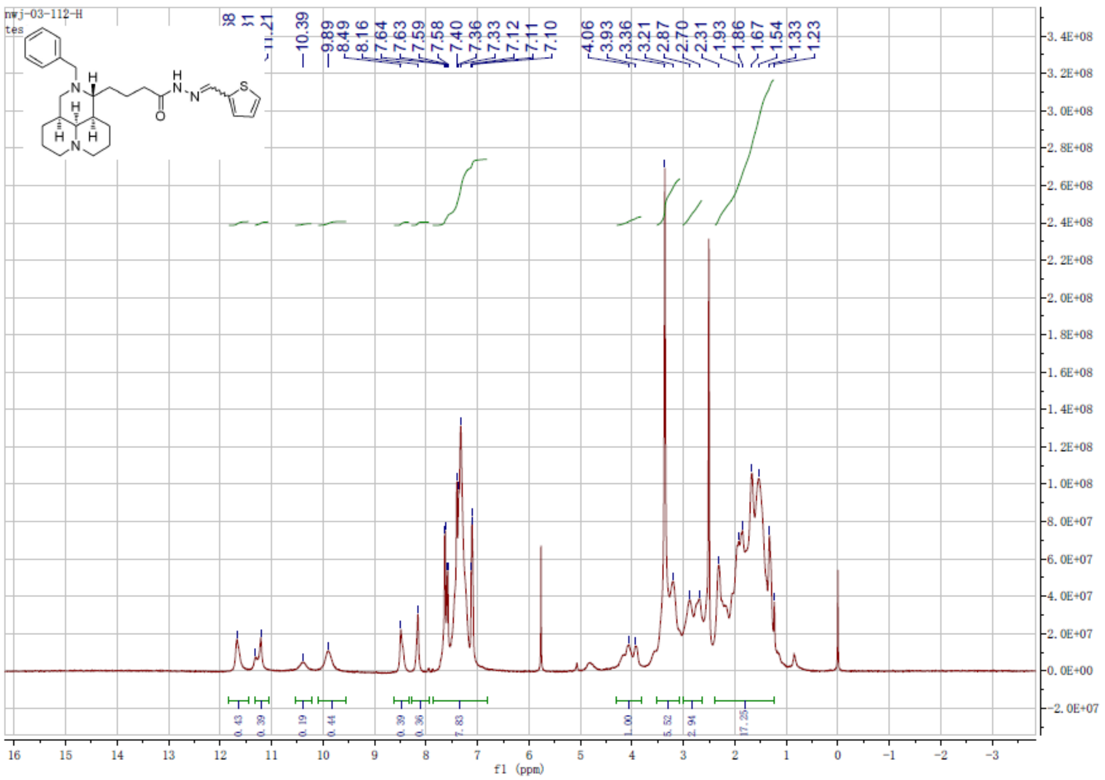

^{1}H NMR of 40

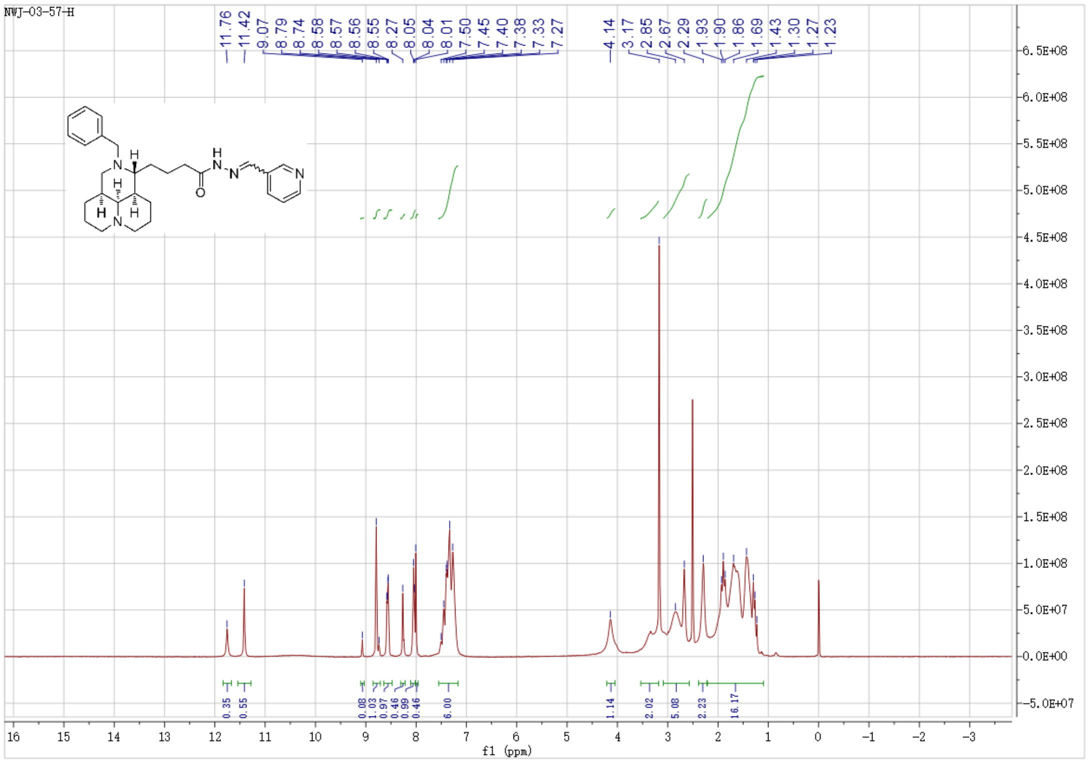

^{1}H NMR of 41

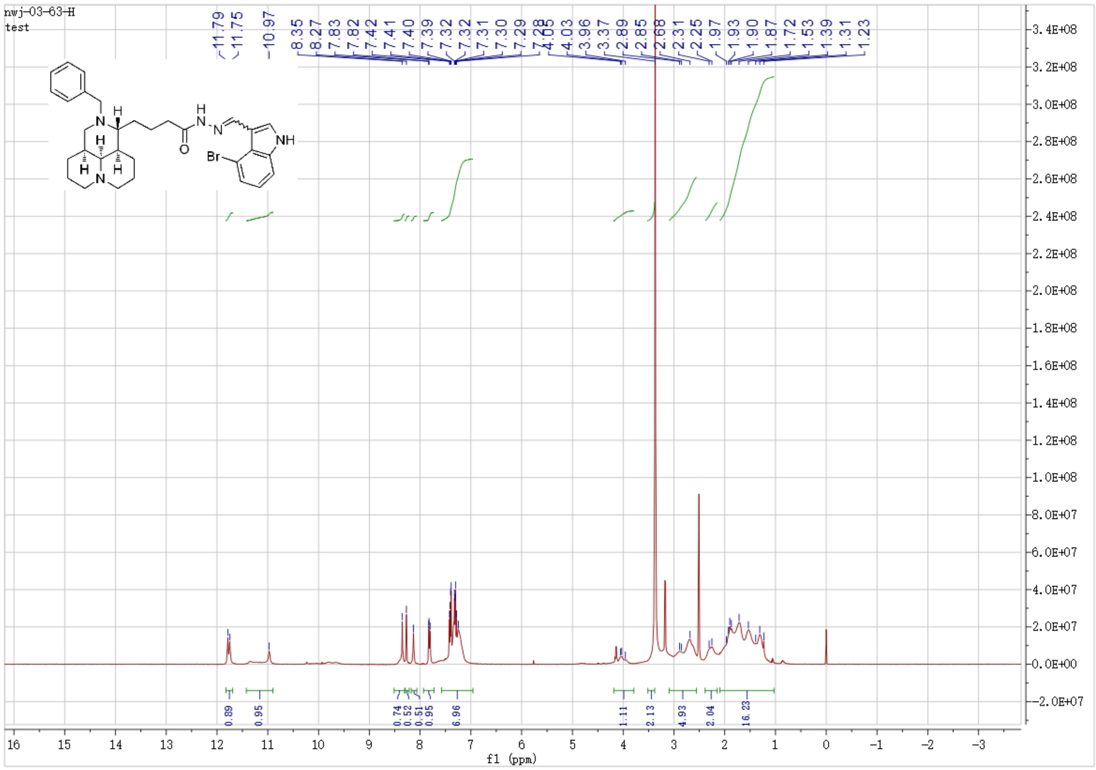

^{1}H NMR of 42

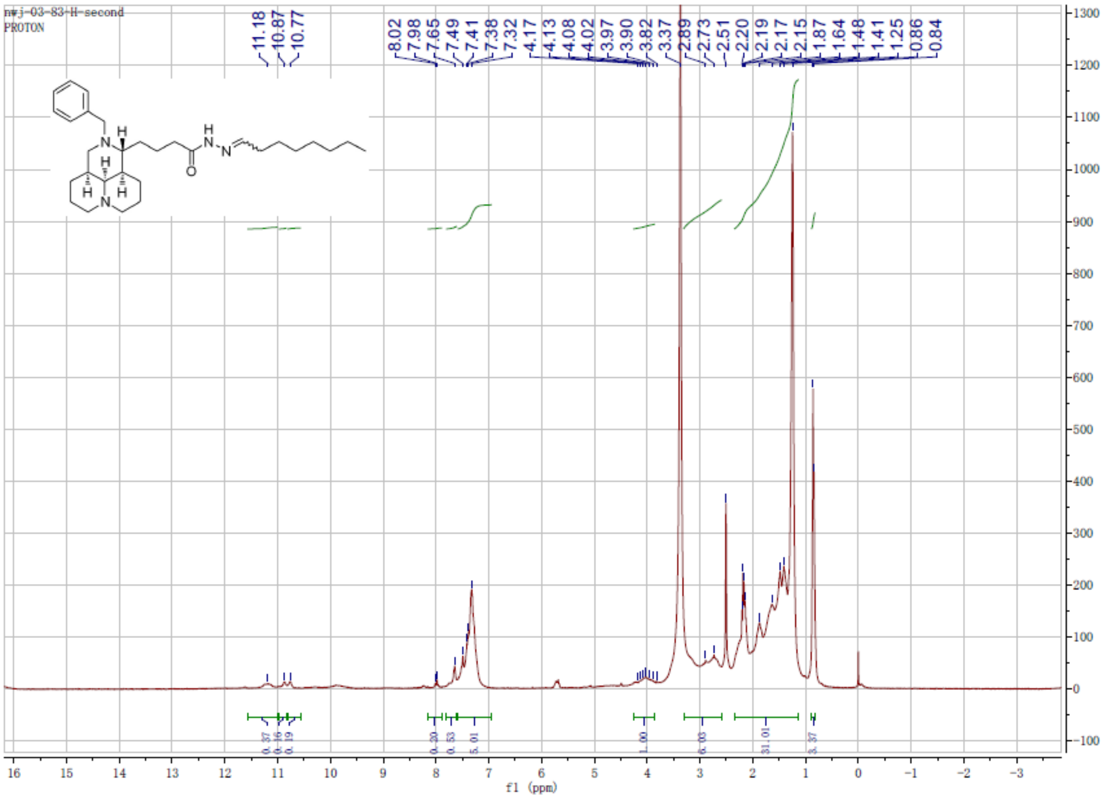

^{1}H NMR of 43

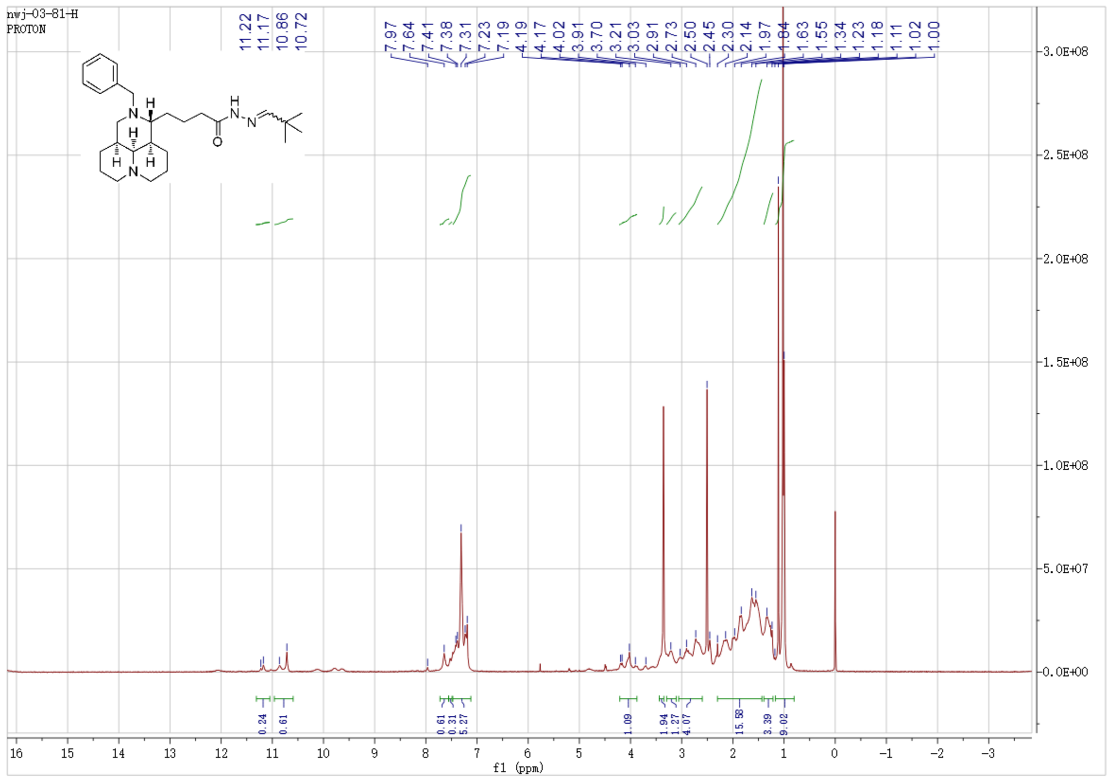

^{1}H NMR of 44

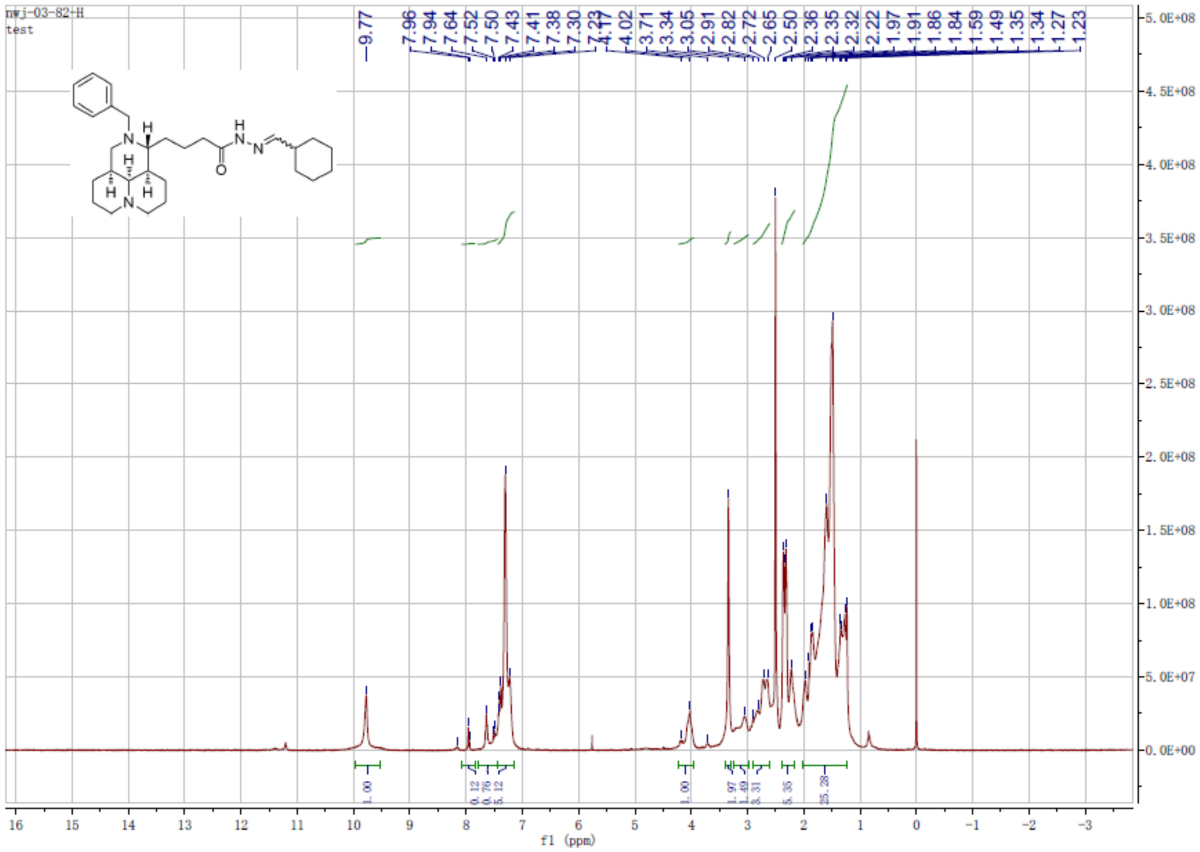

^{1}H NMR of 45

3. Bioassay methods and results for partial insecticidal activities and fungicidal activities.

Detailed bioassay procedures for the anti-TMV activities

Compound solution preparation: Test compound was dissolved within a suitable amount of *N*,*N*-dimethylformamide and diluted with water containing 0.1% TW-80 to make a concentration of 500 μg/mL, and the aqueous solution was diluted to 100 μg/mL.

Antiviral Activity of Compounds against TMV in Vitro. Fresh leaf of the 5−6 growth stage of tobacco (N. tabacum L.) inoculated by the juice-leaf rubbing method (concentration of TMV was 6×10^{-3} μg/mL) was cut into halves along the main vein. The halves were immersed into the solution of test compounds and solvent (double-distilled water containing 0.1% TW-80) for 30 min, respectively, and then cultured at 25 °C for 72 h. The local lesion numbers were then counted.

Inactivation Effect of Compounds against TMV in Vivo. The virus was inhibited by mixing with the compound solution at the same volume for 30 min. The mixture was then inoculated on the growing leaves of the same ages, whereas another pot was inoculated with the mixture of solvent and the virus for control. The local lesion numbers were recorded 3−4 days after inoculation. There were three replicates for each compound.

Curative Effect of Compounds against TMV in Vivo. TMV (concentration of 6.0×10^{-3} mg/mL) was inoculated on the growing leaves of N. tabacum L. of the same ages. Then, the leaves were washed with water and dried. The compound solution was smeared on the leaves, whereas another pot was smeared with solvent for control. The local lesion numbers were then counted and recorded 3−4 days after inoculation. There were three replicates for each compound.

Protective Effect of Compounds against TMV in Vivo. The compound solution was smeared on the growing N. tabacum L. leaves of the same ages. Another pot was smeared with solvent for control. After 12 h, the leaves were inoculated by the juice-leaf rubbing method and then washed with water. The local lesion numbers appearing 3−4 days after inoculation were counted. There were three replicates for each compound.

The in vitro and in vivo inhibition rates of the compound were then calculated according to the following formula (“av” means average, and controls were not treated with compound):

inhibition rate (%) = [(av local lesion number of control − av local lesion number of drugtreated)/av local lesion number of control] × 100%.

Detailed bioassay procedures for the insecticidal activities

Larvicidal Activities against oriental armyworm (Mythimna separata), diamondback moth (Plutella xylostella), cotton bollworm (Helicoverpa armigera), and corn borer (Ostrinia nubilalis): Stock solutions of each test compound was prepared in dimethylformamide at a concentration of 600 mg/L and then diluted to the required concentration (200, 100, and 50 mg/L) with water containing TW-20.

Leaf-dip method was used. Leaf discs (5 cm × 3 cm) were cut from fresh cabbage leaves (or other leaves) and then dipped into the test solution for 3 s. After air-drying, the treated leaf discs were placed individually into vertical tube (or Petri dishes) and the discs were infested with 10 larvae (for example: 10 second-instar diamondback moth larvae, 10 fourth-instar oriental armyworm larvae). Percentage mortalities were evaluated 3 days after treatment.

Evaluations were based on a percentage scale of 0–100, where 0 equals no activity and 100 equals total kill. Each treatment was performed at least two times.

Larvicidal Activities against Mosquito (Culex pipiens pallens). Twenty fourth-instar mosquito larvae were put into the 10 mL of the test solution (concentration 10, 5, and 2 mg/L). Percentage mortalities were evaluated 8 days after treatment.

Evaluations were based on a percentage scale of 0–100, where 0 equals no activity and 100 equals total kill. Each treatment was performed at least two times.

Aphicidal activity against aphid (Aphis Medicnginis Koch): About 60 aphids were transferred to the shoot with 3-5 fresh leaves of horsebean. The shoot with aphids was cut and dipped into the solution of 600 μg/mL of test compound for 2 s, after removing extra solutions on the leaf; the aphids were raised in the shoot for 1 day. Percentage mortalities were evaluated by roughly counting the number of live and dead aphids. Evaluations were based on a percentage scale of 0–100, where 0 equals no activity and 100 equals total kill, and mortality rates were corrected using Abbott's formula. Each treatment was performed at least two times..

Acaricidal activity against adult spider mite (Tetranychus cinnabarinus): Sieva bean plants (*Phaseolus vulgaris* L.) when its primary leaves expanded to 10 cm were selected and cut back to

one plant per pot. Adult mites were moved using a fine brush from the main colony to leaves of the test plants; each leaf was let to have about 60−100 adults. The mite-infested leaves were dipped into the test solution for 3 s with agitation, excess liquid was got rid of by shaking, and then the leaves were placed in a tube (10 cm inner diameter) lined with a piece of filter paper. Percentage mortalities were evaluated 4 days after test by roughly counting the number of live and dead adults. Evaluations were based on a percentage scale of 0–100, where 0 equals no activity and 100 equals total kill, and mortality rates were corrected using Abbott's formula. Each treatment was performed three times.

Detailed bioassay procedures for the fungicidal activities

The compounds were evaluated in mycelial growth tests in artificial media against 14 plant pathogens at rate of 50 mg/L.

Test compound was dissolved within a suitable amount of acetone and diluted with water containing 0.1% TW-80 to the concentration of 500 mg/L. To each petri dish was added 1 mL such solution and 9 mL culture medium to make a 50 mg/L of medicated tablet, whereas to another petri dish was added 1 mL sterilized water and 9 mL culture medium as blank control. A diameter of 4 mm of hyphae was cut by a hole puncher along the hyphae for bacteria to the outer plate and moved to the medicated tablet. Each treatment was performed three times. The dishes were stored in controlled environment cabinets (24±1°C) for 48 h, after which the diameter of mycelia growth was investigated and percentage inhibition was calculated.

Percentage inhibition (%) = (averaged diameter of mycelia in blank controls − averaged diameter of mycelia in medicated tablets) / averaged diameter of mycelia in blank controls

4. Insecticidal activity

Table S1. Larvicidal Activities against Cotton Bollworm (*Helicoverpa Armigera*), Corn Borer (*Ostrinia Nubilalis*), Oriental Armyworm (*Mythimna Separate*), aphicidal activity against aphid (*Aphis Medicnginis* Koch), and acaricidal activity against spider mite (*Tetranychus cinnabarinus*) of Compounds **1-45**.

Compd.	mortality (%) at 600 μg/mL				
	H. Armigera	*O. Nubilalis*	*M. Separate*	*A. Medicnginis* Koch	*T. cinnabarinus*
**1**	40±10	0	0	0	0
**2**	0	0	0	0	0
**3**	0	0	0	0	40
**4**	0	0	0	0	0
**5**	0	0	0	0	0
**6**	0	0	0	0	0
**7**	0	0	10	0	0
**8**	0	10±10	0	30±10	0
**9**	0	0	20±10	0	0
**10**	20±10	20±0	0	0	0
**11**	40±0	30	30±0	0	0
**12**	0	0	0	30±0	0
**13**	40±10	35±5	20±10	20±0	0
**14**	0	20	10±0	0	0
**15**	0	0	0	0	20±10
**16**	40±10	10±10	0	0	0
**17**	20±0	0	0	0	0
**18**	0	0	0	0	30±0
**19**	0	40±10	20±10	0	0
**20**	60±10	0	0	20±10	0
**21**	0	50±	20±10	0	0

Compd.	mortality (%) at 600 μg/mL				
	H. Armigera	*O. Nubilalis*	*M. Separate*	*A. Medicnginis* Koch	*T. cinnabarinus*
**22**	40±10	0	40±0	0	20±0
**23**	0	30±0	20±10	0	0
**24**	60±0	20±10	40±10	0	0
**25**	20±0	0	0	0	0
**26**	10±10	15±5	20±0	0	0
**27**	25±5	35±5	20±0	0	0
**28**	5±5	10±10	20±0	0	0
**29**	5±5	15±5	0	0	0
**30**	0	0	0	0	0
**31**	10±0	15±5	20±10	0	0
**32**	0	0	0	0	0
**33**	15±5	20±10	0	0	0
**34**	0	0	0	0	0
**35**	5±5	5±5	0	0	0
**36**	10±0	0	10±10	0	0
**37**	20±10	0	20±0	0	0
**38**	15±5	20±10	20±10	0	0
**39**	0	0	0	0	30±0
**40**	5	5±5	0	0	20±0
**41**	0	0	0	0	0
**42**	0	10±0	0	0	0
**43**	0	0	0	0	0
**44**	0	0	0	0	0
**45**	0	0	0	0	0

5. Fungicidal activity

Table S2. Fungicidal activity of compounds **1-45** against fourteen kinds of phytopathogens[a]

Compd	fungicidal activity (%) at 50 μg/mL													
	F.C.	*C.H.*	*P.P.*	*R.C.*	*B.M*	*C.O.*	*F.M.*	*A.S.*	*F.G.*	*P.I.*	*P.C.*	*S.S.*	*B.C.*	*R.S.*
Chlorothalonil	73±1	81±2	74±1	96±2	96±2	96±1	83±2	97±1	35±2	97±1	98±1	97±1	94±2	96±1
Carbendazim	97±1	35±2	97±1	98±1	97±1	94±2	96±1	65±2	38±1	88±2	58±2	80±1	65±3	96±1
matrine	25±2	23±1	31±2	44±1	74±1	54±1	43±1	5±2	7±1	30±1	12±2	8±1	10±1	13±3
**1**	14±2	24±1	21±2	36±1	13±1	10±2	13±1	13±1	24±1	6±1	25±1	20±1	14±2	12±1
**2**	14±1	21±1	45±1	28±1	13±1	23±1	19±1	27±1	15±1	13±1	36±1	18±1	14±1	6±1
**3**	30±1	28±2	48±1	74±1	18±1	26±1	48±1	27±1	27±1	44±1	46±1	26±1	22±2	16±1
**4**	19±1	38±2	36±1	78±1	13±1	23±1	29±1	40±1	21±1	13±1	29±1	45±1	47±2	7±1
**5**	26±1	31±2	38±1	32±1	18±1	23±2	16±1	27±1	33±1	13±1	32±2	26±1	22±2	23±2
**6**	30±1	24±1	34±1	48±1	26±2	23±1	19±1	33±1	30±1	19±2	25±1	20±1	28±2	9±1
**7**	14±1	31±1	23±1	46±1	18±2	26±1	29±1	27±2	30±1	19±2	11±2	20±2	11±1	6±2
**8**	28±2	35±1	36±2	44±1	13±1	19±1	23±2	27±2	42±2	31±1	61±2	59±1	47±1	6±1
**9**	19±2	17±1	27±1	24±3	8±1	23±2	16±1	20±1	15±1	13±3	54±2	33±1	14±1	25±2
**10**	16±1	38±1	48±1	70±2	11±2	23±2	16±1	27±2	12±1	19±1	25±1	20±2	36±1	6±1
**11**	16±1	17±1	16±1	31±1	11±2	29±1	3±1	13±1	21±1	13±2	29±2	13±1	28±2	6±1
**12**	16±1	28±2	34±1	42±1	18±1	29±1	23±2	27±2	27±1	25±1	43±1	20±2	28±1	16±1
**13**	26±1	24±1	30±1	21±1	8±1	16±1	13±1	27±1	12±1	13±1	18±1	29±1	11±1	6±1
**14**	28±1	24±1	54±2	46±2	21±1	16±1	19±1	33±1	36±2	25±1	39±1	20±1	19±3	25±2
**15**	33±1	41±2	66±1	80±1	32±2	42±1	32±1	33±1	42±1	38±1	64±1	24±2	36±1	62±2
**16**	35±1	52±1	52±1	82±1	24±1	33±1	42±1	20±1	30±1	25±1	29±2	13±1	14±1	6±1

Compd	fungicidal activity (%) at 50 μg/mL													
	F.C.	*C.H.*	*P.P.*	*R.C.*	*B.M*	*C.O.*	*F.M.*	*A.S.*	*F.G.*	*P.I.*	*P.C.*	*S.S.*	*B.C.*	*R.S.*
**17**	21±1	21±1	34±1	40±1	8±2	16±1	13±2	20±2	12±1	13±3	11±2	26±1	14±1	6±1
**18**	19±2	24±1	34±1	33±1	11±3	13±1	13±1	20±1	21±1	13±2	21±2	24±1	11±1	6±1
**19**	19±2	17±1	32±1	30±2	11±2	16±1	3±2	20±1	21±1	6±1	25±1	20±2	17±2	6±1
**20**	16±1	24±1	18±1	38±2	16±2	13±2	13±1	7±3	18±1	13±1	21±2	28±2	14±1	12±1
**21**	33±2	52±1	54±1	74±1	24±2	23±2	42±1	33±1	39±1	25±1	43±1	20±2	28±1	31±1
**22**	23±1	24±1	36±1	31±1	18±2	19±1	16±1	20±1	15±1	13±2	25±1	13±1	23±2	12±1
**23**	21±1	24±1	29±2	33±1	11±2	29±1	13±1	20±1	21±1	19±1	14±1	20±2	14±1	5±2
**24**	23±1	21±1	30±1	33±1	13±1	16±1	7±3	20±1	21±1	19±1	14±1	24±2	17±2	19±3
**25**	21±1	28±1	88±1	46±1	16±2	26±1	52±2	33±1	30±1	25±1	57±1	26±1	14±2	15±2
**26**	11±1	17±1	58±1	12±2	10±12	13±2	17±1	8±1	19±2	6±2	6±2	6±3	8±2	4±2
**27**	16±1	14±2	38±2	9±1	8±12	16±1	17±3	8±2	23±2	6±2	19±2	15±2	13±3	6±1
**28**	14±2	24±1	58±2	23±3	8±2	16±1	13±3	17±2	10±2	6±2	6±2	6±2	21±1	2±2
**29**	14±2	28±2	45±2	23±2	13±2	16±1	54±1	8±2	16±1	6±1	13±3	24±1	17±2	12±2
**30**	16±2	24±1	62±2	16±1	15±2	26±1	8±2	8±2	29±1	6±1	31±2	24±1	21±1	6±2
**31**	11±1	35±2	46±1	58±1	8±1	19±1	25±1	25±1	42±1	13±2	31±1	6±2	8±1	6±1
**32**	30±1	28±2	32±2	29±1	21±2	19±2	29±1	8±3	23±2	13±3	31±2	39±1	33±2	6±1
**33**	22±2	62±1	45±2	47±3	31±1	19±2	0±0	42±1	61±2	50±2	75±1	43±2	46±1	16±2
**34**	32±2	35±2	51±2	33±2	13±1	23±2	21±2	16±2	45±1	31±2	75±2	52±2	38±3	41±2
**35**	11±1	17±2	53±1	23±2	15±3	16±2	13±2	8±2	16±2	6±2	6±2	33±2	29±1	6±2
**36**	11±2	24±1	38±2	22±1	18±1	13±1	0±0	42±2	55±1	38±2	81±1	44±2	33±2	69±1
**37**	14±3	21±2	31±1	17±2	10±2	16±1	16±2	17±2	36±3	6±2	63±2	30±3	13±3	2±3

Compd	fungicidal activity (%) at 50 μg/mL													
	F.C.	*C.H.*	*P.P.*	*R.C.*	*B.M*	*C.O.*	*F.M.*	*A.S.*	*F.G.*	*P.I.*	*P.C.*	*S.S.*	*B.C.*	*R.S.*
**38**	16±2	31±1	37±2	28±1	18±2	23±2	25±2	8±2	16±2	6±2	6±2	15±2	17±2	61±1
**39**	14±2	21±2	24±2	28±1	18±1	23±2	17±2	25±2	16±1	31±1	31±1	43±2	38±3	57±1
**40**	30±2	59±1	95±2	81±2	36±1	36±2	29±1	8±2	19±2	6±3	6±2	30±2	13±1	12±2
**41**	16±2	17±2	49±3	8±2	13±2	19±1	21±2	33±1	16±2	13±2	31±2	61±2	17±2	22±2
**42**	22±2	28±3	0±0	52±2	18±2	13±2	17±2	25±2	26±2	6±3	31±2	33±3	12±2	20±2
**43**	14±2	24±1	38±1	49±1	10±2	10±2	21±1	33±2	16±2	13±2	63±3	43±3	42±2	27±3
**44**	8±2	31±1	54±1	35±1	15±2	16±1	4±3	8±3	45±2	13±2	3±2	50±2	33±3	4±2
**45**	19±2	28±2	47±2	12±2	8±2	10±2	8±2	8±2	23±2	6±2	13±1	30±2	25±1	4±2

[a]*F.C.*, *Fusarium oxysporum sp. cucumeris*; *C.H.*, *Cercospora arachidicola Hori*; *P.P.*, *Physalospora piricola*; *R.C.*, *Rhizoctonia cerealis*; *B.M.*, *Bipolaris maydis*; *C.O.*, *Colletotrichum orbiculare*; *F.M.*, *Fusarium moniliforme*; *A.S.*, *Alternaria solani*; *F.G.*, *Fusarium graminearum*; *P.I.*, *Phytophthora infestans*; *P.C.*, *Phytophthora capsici*; *S.S.*, *Sclerotinia sclerotiorum*; *B.C.*, *Botrytis cinerea*; *R.S.*, *Rhizoctonia solani*. Color in red means that the inhibition data are above 70%.

6. References

S1. Ni, W. J.; Li, C. J.; Liu, Y. X.; Song, H. J.; Wang, L. Z.; Song, H. B.; Wang, Q. M. Various Bioactivity and Relationship of Structure–Activity of Matrine Analogues. J. Agric. Food Chem. 2017, 65 (10), 2039–2047.

S2. He, L. Q.; Gu, H. X.; Yin, D. K.; Zhang, Y. H.; Wang, X. S. Synthesis and biological evaluation of nitric oxide-releasing matrine derivatives as anticancer agents. Chem. J. Chin. Univ. 2010, 31, 1541−1547.
